# *Lactobacillus lactis* and *Pediococcus pentosaceus*‐driven reprogramming of gut microbiome and metabolome ameliorates the progression of non‐alcoholic fatty liver disease

**DOI:** 10.1002/ctm2.634

**Published:** 2021-12-29

**Authors:** Jeong Seok Yu, Gi Soo Youn, Jieun Choi, Chang‐Ho Kim, Byung Yong Kim, Seung‐Jo Yang, Je Hee Lee, Tae‐Sik Park, Byoung Kook Kim, Yeon Bee Kim, Seong Woon Roh, Byeong Hyun Min, Hee Jin Park, Sang Jun Yoon, Na Young Lee, Ye Rin Choi, Hyeong Seob Kim, Haripriya Gupta, Hotaik Sung, Sang Hak Han, Ki Tae Suk, Do Yup Lee

**Affiliations:** ^1^ Department of Agricultural Biotechnology Center for Food and Bioconvergence Research Institute for Agricultural and Life Sciences Seoul National University Seoul Republic of Korea; ^2^ Institute for Liver and Digestive Diseases Hallym University Chuncheon Republic of Korea; ^3^ ChunLab, Inc. Seoul Republic of Korea; ^4^ Department of Life Science Gachon University Sungnam Republic of Korea; ^5^ Chong Kun Dang Bio Research Institute Gyeonggi‐do Republic of Korea; ^6^ Microbiology and Functionality Research Group World Institute of Kimchi Gwangju Republic of Korea; ^7^ School of Medicine Kyungpook National University Daegu Republic of Korea; ^8^ Department of Pathology Hallym University College of Medicine Chuncheon Republic of Korea

**Keywords:** gut‐liver axis, indole, metabolites, microbiome, non‐alcoholic fatty liver disease

## Abstract

**Background:**

Although microbioa‐based therapies have shown putative effects on the treatment of non‐alcoholic fatty liver disease (NAFLD), it is not clear how microbiota‐derived metabolites contribute to the prevention of NAFLD. We explored the metabolomic signature of *Lactobacillus lactis* and *Pediococcus pentosaceus* in NAFLD mice and its association in NAFLD patients.

**Methods:**

We used Western diet‐induced NAFLD mice, and *L. lactis* and *P. pentosaceus* were administered to animals in the drinking water at a concentration of 10^9^ CFU/g for 8 weeks. NAFLD severity was determined based on liver/body weight, pathology and biochemistry markers. Caecal samples were collected for the metagenomics by 16S rRNA sequencing. Metabolite profiles were obtained from caecum, liver and serum. Human stool samples (healthy control [*n* = 22] and NAFLD patients [*n* = 23]) were collected to investigate clinical reproducibility for microbiota‐derived metabolites signature and metabolomics biomarker.

**Results:**

*L. lactis* and *P. pentosaceus* supplementation effectively normalized weight ratio, NAFLD activity score, biochemical markers, cytokines and gut‐tight junction. While faecal microbiota varied according to the different treatments, key metabolic features including short chain fatty acids (SCFAs), bile acids (BAs) and tryptophan metabolites were analogously restored by both probiotic supplementations. The protective effects of indole compounds were validated with in vitro and in vivo models, including anti‐inflammatory effects. The metabolomic signatures were replicated in NAFLD patients, accompanied by the comparable levels of *Firmicutes*/*Bacteroidetes* ratio, which was significantly higher (4.3) compared with control (0.6). Besides, the consequent biomarker panel with six stool metabolites (indole, BAs, and SCFAs) showed 0.922 (area under the curve) in the diagnosis of NAFLD.

**Conclusions:**

NAFLD progression was robustly associated with metabolic dys‐regulations in the SCFAs, bile acid and indole compounds, and NAFLD can be accurately diagnosed using the metabolites. *L. lactis* and *P. pentosaceus* ameliorate NAFLD progression by modulating gut metagenomic and metabolic environment, particularly tryptophan pathway, of the gut‐liver axis.

## Graphical abstract

Diet with the *L. lactis* and *P. pentosaceus* inhibited NAFLD progression via normalization of metabolites including indoles, SCFAs, or BA composition, which in turn was achieved by modulation of gut microbiota composition and production of anti‐inflammatory metabolites.

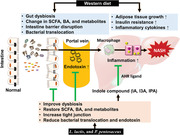

## 1 INTRODUCTION

Non‐alcoholic fatty liver disease (NAFLD) is one of the most common causes of chronic liver disease worldwide, and its incidence is constantly growing given its association with obesity, coronary artery disease, metabolic syndrome and diabetes. NAFLD encompasses fatty liver and non‐alcoholic steatohepatitis (NASH), and currently lifestyle change involving dietary regulation and exercise is the only therapeutic option for NAFLD.^1^ Although many drugs have been developed for NAFLD, none of them have been approved by the US Food and Drug Administration due to difficulties in patient compliance and failure in clinical trials.^2^

Recent studies have reported that gut microbiota is implicated in liver disease along the gut‐liver axis. Gut microbiota itself and metabolites produced by microbiota—known as postbiotics—are closely related to host homeostasis, and the compositional changes have shown crucial roles in various human diseases.^3^, ^4^ Short chain fatty acids (SCFAs) are the best‐known gut microbiota‐derived metabolites, which have shown important roles in the maintenance of health and disease development.^5^ More recently, tryptophan metabolites have been increasingly recognized as a new therapeutic, which reduces the production of pro‐inflammatory cytokines by downregulating macrophages, scavenging free radicals and reducing oxidative stress.^4^, ^6^

Our previous report proposed that *Lactobacillus* and *Pediococcus* supplementation improved NAFLD by modulating the gut microbiota and inflammation.^7^ Other studies have also demonstrated that lactic acid bacteria are effective in the liver disease.^8^, ^9^ However, microbiota‐derived metabolomic signatures of *L. lactis* and *P. pentosaceus* were not clearly defined in the prevention of NAFLD.

Accordingly, our current study aimed to interrogate the underlying mechanism of the probiotics, which were approved for human use (Table S1) and showed promising results in the prevention of NAFLD (Figure S1) following the previous study. Consequently, we discovered keystone metabolites that were causatively implicated in molecular, biochemical and pathological parameters for the prevention of NAFLD. Besides, we proposed highly discriminative biomarker panel based on comparative analysis of the gut metabolomic signatures of Western diet (WD)‐induced NAFLD mice and patients with NAFLD.

## 2 METHODS

### 2.1 Human data

A prospective cohort study was carried out between April 2017 and March 2020 (ClinicalTrials.gov NCT04339725). This study involved patients with liver disease who were followed‐up at the hepatology department of Hallym University. For the NAFLD patient group, patients with elevated liver enzyme (aspartate aminotransferase [AST] or alanine aminotransferase [ALT] ≥ 50 IU/L) and older than 20 years were included. Enrolled patients did not drink excessive alcohol (male > 60 and female > 40 g/week) for the study. The exclusion criteria were as follows: patients with viral hepatitis, alcoholic hepatitis, autoimmune hepatitis, pancreatitis, hemochromatosis, Wilson's disease, drug‐induced liver injury and cancers. We excluded patients taking drugs that affect the gut microbiota at enrollment. For the control group, we enrolled normal populations who come to the hospital for health check‐up.

This study was conducted in accordance with the ethical guidelines (1975 Helsinki Declaration), as reflected by a prior approval by the institutional review board for human research in hospitals participating in the study (2016–134). Informed consent was obtained from all participants.

### 2.2 Experimental animals

Male C57BL/6J mice (6 weeks old) were purchased by Dooyeol Biotech (Seoul, Korea). All mice were maintained in a cage of five mice with a constant environment (22 ± 2°C with 12/12‐h light/dark cycle). Mice received human care and monitored daily. For the adaptation period, mice were fed each diet for 1 week. The WD (TD88137, Seoul, Korea) was purchased, and the composition of nutrients was 42% fat, 42.7% of carbohydrate and 15% protein. Animal studies were approved by the Institutional Animal Care and Use Committee of the College of Medicine, Hallym University (2018–04, 45 and 60).

### 2.3 Stool analysis for 16S rRNA amplicon sequencing

Human faeces were stored at −20°C as soon as the patient received 2–3 g of faeces using the kit (stool paper and stool box) and moved to −80°C within 1 day. For mice faeces, whole intestine was collected during euthanizing and frozen at −80°C and stored. Genomic DNA for metagenomic sequencing was extracted with a QIAamp stool kit (Qiagen, Germany), and library was prepared with an NEBNext Ultra II FS DNA Library Prep Kit for Illumina (New England BioLabs, USA) according to the manufacturer's directions. The quantification of libraries was checked using a Qubit dsDNA HS assay kit (ThermoFisher Scientific, USA) and confirmed by qPCR with KAPA SYBR FAST qPCR Master Mix kit (Kapa Biosystems, USA). The quality of libraries was assessed on a Bioanalyzer 2100 (Agilent, USA) using a DNA 12000 chip. All libraries were sequenced on the NovaSeq 6000 platform (Illumina, USA) with a paired‐end (PE) 150 bp reads.

The analysis was performed following our previous method.^10^ In brief, DNA was extracted with a QIAamp stool kit, and amplification of the V3‐V4 region of the bacterial 16S rRNA gene was conducted using barcoded fusion primers. The forward fusion primer contained p5 adapter, i5 index and gene‐specific primer 341F (5′‐AATGATACGGCGACCACCGAGATCTACAC‐XXXXXXXX‐TCGTCG GCAGCGTCAGATGTGTATAAGAGACAG‐CCTACGGGNGGCWGCAG‐3′; underlining indicates the target region primer, and X indicates the barcode region), and the reverse fusion primer contained p7 adapter, i7 index and gene‐specific primer 805R (5′‐CAAGCAGAAGACGGCATACGAGAT‐XXXXXXXXGTCTCGTGGGCTCGGAGATGTGTATAAGAGACAG‐GACTACHVGGGTATCT AATCC‐3′), in which included sequencing adapters and dual‐index barcodes of the Nextera XT kit (Illumina, San Diego, CA, USA). The index sequences used per sample are provided as Table S2. The amplification was performed in the C1000 touch thermal cycler polymerase chain reaction system (Bio‐Rad Laboratories, Inc., USA) with the following conditions: initial denaturation of 3 min at 95°C; followed by 25 cycles of denaturation at 95°C for 30 s, annealing at 55°C for 30 s, extension at 72°C for 30 s and final extension at 72°C for of 5 min. Each amplified PCR product was confirmed with 1% agarose gel electrophoresis and visualized on a Gel Doc XR+ imaging system (Bio‐Rad laboratories, Inc., USA). The amplified products were purified and size‐selected by Agencourt AMPure XP beads (Beckman Coulter, USA). Library was constructed with pooled PCR products, and quality of library was assessed on a Bioanalyzer 2100 (Agilent, USA) using a DNA 12000 chip and quantified by qPCR with KAPA SYBR FAST qPCR Master Mix kit (Kapa Biosystems, USA). Sequencing was carried out according to the manufacturer's instructions at Chunlab, Inc. (Seoul, Republic of Korea) with the Illumina MiSeq platform using reagent kit V3 in PE 250 bp mode. Microbiome taxonomic profiling (MTP) was conducted with the EZBioCloud platform (ChunLab Inc., Republic of Korea) using the database version PKSSU4.0. After taxonomic profiling of each sample, comparative microbiome taxonomic profiling analyzer of EZBioCloud was used for the comparative analysis of the samples. The number of operational taxonomic units picking was conducted with UCLUST and CDHIT with 97% of similarity cutoff.^11^ Subsequently, Good's coverage, rarefaction and alpha‐diversity indices including ACE, Chao1, Jackknife, Shannon, Simpson and NPShannon were calculated. Beta‐diversity was shown by clustering using the unweighted pair group method with arithmetic mean and principal coordinate analysis (PCoA).

### 2.4 Metabolic profiling of mouse samples (caecum, serum and liver) and human stool samples

The metabolomic profiles of mouse caecum were acquired using a combination of gas chromatography‐mass spectrometry (GC‐MS) and two liquid chromatography‐mass spectrometry (LC–MS) methods. Caecal samples were thawed at 4°C and mixed with 1.1 ml of cold extraction solvent I (acetonitrile/water 1:1, v/v). The mixtures were vortexed for 1 min and sonicated for 5 min under ice and centrifuged at 13 200 rpm for 5 min at 4°C. Each supernatant (500 μl) was transferred into a new 2 ml tube for SCFAs analysis (Method 1, see the supplementary method for details).^12^ The rest of supernatant was mixed with 600 μl of cold extraction solvent II (acetonitrile/methanol, 1:3, v/v). For the second extraction step, the mixtures were vortexed for 1 min and centrifuged at 13 200 rpm for 5 min at 4°C. The supernatants (500 μl) were aliquoted and transferred to new 1.5‐ml tubes for gas‐chromatography time‐of‐flight mass spectrometry (Method 2) and liquid‐chromatography Orbitrap mass spectrometry (Method 3). The aliquots were concentrated to complete dryness using a speed vacuum concentrator (SCANVAC, Korea).^13^ Hepatic and serum indoles were analyzed based on Method 3. Similarly, human stool metabolomic profiles were obtained based on Methods 1, 2 and 3. The caecal data acquired by Gas chromatography‐time‐of‐flight mass spectrometry (GC‐TOF MS) have been retrieved from our previous study.^7^

#### 2.4.1 Method 1: SCFAs analysis using LC‐Orbitrap MS

The supernatant (40 μl) of extraction solution I (acetonitrile/water, 1:1, v/v) was mixed with 20 μl of 200 mM 3‐nitrophenylhydrazine‐HCL in acetonitrile (70 %) and 20 μl of a 1‐ethyl‐3‐(3‐dimethylaminopropyl) carbodiimide‐HCL (120 mM) dissolved in 6 % pyridine solution. The mixture was incubated for 30 min at 40°C and diluted with 1.92 ml of 70 % of acetonitrile.^12^

The diluted derivatives were chromatographically separated with a 150 × 2.1 mm ultra high performance liquid chromatography ethylene bridged hybrid (UPLC BEH) 1.7‐μm C18 column (Waters, Milford, MA, USA) equipped with 5.0 mm × 2.1 mm UPLC BEH 1.7 μm C18 VanGuard Pre‐Column (Waters) controlled by Ultimate‐3000 UPLC system (Thermo Fisher Scientific, Waltham, MA, USA). The mobile phase composed of 0.01% formic acid in water (buffer A, v/v) and 0.01% formic acid in acetonitrile (buffer B, v/v) with a flow rate of 0.3 ml/min. The gradient of LC elution was programmed as follows: equilibration in 15% buffer B for 2 min, 15%–55 % buffer B gradient over 9 min, 100 % buffer B held for 1 min and re‐equilibration in 15% buffer B for 3 min. Injection volume was 2 μl for both MS1 and MS/MS analysis. Mass spectra were acquisition using Q‐Exactive Plus Orbitrap (Thermo Fisher Scientific, Waltham) equipped with an electrospray ionization (ESI) interface (HESI‐II) in negative ionization, and the system was controlled using Xcalibur 4.0 and Q‐Exactive Tune software. Raw data were processed by Tracefinder software (version 4.0, Thermo Fisher Scientific, San José, CA, USA). A mass tolerance for precursor ion and retention time tolerance were set to 5 ppm and 0.5 min, respectively.

#### 2.4.2 Method 2: Primary metabolites profiling using GC‐TOF MS

The dried metabolites were derivatized with 5 μl of 40 mg/ml methoxyamine hydrochloride (Sigma‐Aldrich, St. Louis, MO, USA) dissolved in pyridine (Sigma‐Aldrich, St. Louise, MO, USA) for 90 min at 30°C. After the first derivatization step, the second derivatization step was done with 45 μl of N‐methyl‐N‐trimethylsilyltrifluoroacetamide (MSTFA +1% TMCS; Restek, Bellefonte, PA, USA) for 1 h 60 min at 37°C. Internal retention time index was added which included 13 fatty acids methyl esters (C8, C9, C10, C12, C14, C16, C18, C20, C22, C24, C26, C28, and C30).^14^

The injection (5 μl) of the derivatized metabolites was programmed by an Agilent 7693 ALS (Agilent Technologies, Wilmington, DE, USA) in splitless mode. GC‐TOF MS analysis was performed using an Agilent 7890B gas chromatograph (Agilent Technologies) and Leco Pegasus HT time of flight mass spectrometer (LECO, St. Joseph, MI, USA). Oven temperature was initiated at 50°C (1 min), gradually increased at 20°C/min to 330°C, and held constant for 5 min. Transfer line and ion source temperatures were set to 280°C and 250°C, respectively. Mass spectra were collected ranging from 85 to 500 m/z at a scan rate of 20 spectra/s with a detector voltage of 1850 V.^15^

Data pre‐processing was conducted using ChromaTOF software (version 4.5), which included apex mass values, full spectrum, peak purity, signal‐to‐noise ratio and retention time. Generic text file (.txt) and netCDF file were produced based on ChromaTOF‐specific Pegasus file (.peg) for peak identification and semi‐quantification. The post‐process was performed using *Binbase* algorithm including chromatogram validation, primary RI detection and validation of unique mass.^16^, ^17^ A peak height of single quant ion was generated for statistical analysis. Missing values that did not pass the primary criteria were imputed by post‐matching and replacement using raw data as previously described.^14^ To evaluate analytical precision, a mixture of 25 pure reference compounds was analyzed every six samples, which demonstrated reproducibility during analysis (Figure S7).

#### 2.4.3 Method 3: Secondary metabolites profiling of LC‐Orbitrap MS

The dried extracts were reconstituted with 50 μl of 70% acetonitrile for LC‐Orbitrap MS analysis. Chromatographic separation was carried out using Ultmate‐3000 UPLC system (Thermo Fisher Scientific, Waltham) and a 150 × 2.1‐mm UPLC BEH 1.7‐μm C18 column (Waters) equipped with 5.0 mm × 2.1‐mm UPLC BEH 1.7 μm C18 VanGuard Pre‐Column (Waters). Mobile phase consisted of buffer A (0.1% formic acid in water) and buffer B (0.1% formic acid in 100% acetonitrile). A flow rate was set to 0.35 ml/min, and a gradient was programmed as follows: equilibration in 3% buffer B for 1 min, 3%–100% buffer B gradient over 9 min, 100% buffer B held for 1 min and re‐equilibration in 3% buffer B for 3 min.

Mass‐spectrometric analysis was performed on a Q‐Exactive plus Orbitrap (Thermo Fisher Scientific, Waltham) with ionization polarity‐switching mode. Full MS scan was conducted on the metabolites (70–1000 m/z) with resolution of 70 000 FWHM at m/z = 200 and with automatic gain control (AGC) target of 1e6 ions and maximum injection time (IT) of 100 ms. Data‐dependent MS/MS analysis was performed on pooled samples by each ionization mode. Data‐dependent MS/MS setting was as follows: Top10 MS1 ions; resolution, 17 500 at 200 m/z; AGC target, 1e5; maximum IT, 50 ms; isolation window, 1.0 m/z; normalized collision energy (NCE), 30; intensity threshold, 2e3 ions; apex trigger, 3–6 s; dynamic exclusion, 6 s. M/Z values and retention times for seven bile acids (BAs) were added to the inclusion list for the application of higher collision energy (NCE, 70).

Data acquisition and pre‐processing were conducted using Xcalibur software (Thermo Fisher Scientific, San José). The obtained RAW data files were processed using Compound Discoverer software (version 2.0, Thermo Fisher Scientific, San José). The data processing was done following the workflow such as Select spectra, Align Retention times, Detect Unknown Compounds, Group Unknown Compounds, Fill Gaps and Search mzCloud. Mass tolerance of MS1 on every node was set at 5 ppm. Align Retention Time node was set to 1 min to Maximum shift. Compound identification was done against mzCloud with criteria of 10 ppm (MS2 mass tolerance) and 70% of assignment threshold.

#### 2.4.4 Indole profiling for mouse liver and serum samples

Indole profiling for mouse liver and blood serum was performed using LC‐Orbitrap MS. Liver samples were weighed and lyophilized for 72 h. The lyophilized livers were ground using Mixer Mill MM400 (Retsch GmbH & Co., Germany) and mixed with 500 μl of cold extraction solvent III (methanol:acetonitrile:water, 3:3:2, v/v/v) per 100 mg of liver sample. The mixtures were sonicated for 10 min and centrifuged for 15 min at 13 200 rpm and 4°C. The supernatants were transferred to new 1.5 ml tubes and dried completely by a speed vacuum concentrator. Mouse serum samples (100 μl) were extracted by 1 ml of cold extraction solvent III, and the remaining extraction procedures were identical to the liver samples. The dried liver and blood extracts were reconstituted with 50 μl of 80% methanol. LC‐Orbitrap MS analysis was conducted based on Method 3.

### 2.5 Bioinformatics

Statistical analyses were conducted on all continuous variables acquired from GC‐MS and LC‐MS. All datasets were normalized using the ‘MS total useful signal.’^18^ Significant differences between two groups were determined by Mann–Whitney *U* test and Student's *t*‐test. A Kruskal–Wallis test with Dunn's post hoc was conducted to evaluate significant differences among four groups using package dunn.test in the software R.^19^ The *p* value was corrected by Benjamini–Hochberg's adjustment (false discovery rate) and pathway over‐representation analysis were performed based on the statistical modules implemented in MetaboAnalyst 4.0 (based on the hypergeometric test and relative‐betweenness centrality).^20^ Treemap and Pie chart were created through Microsoft Excel (Microsoft, Seattle, WA, USA) using compound classification by human metabolome database.^21^ The metabolic network map was constructed based on structural similarity (Tanimoto score) and biochemical liaison (Kyoto encyclopedia of genes and genomes [KEGG] reaction pair information) and visualized by a prefuse force‐directed layout using Cytoscape version 3.7.2.^22^ SIMCA 15 (Umetrics AB, Umea, Sweden) was applied for multivariate statistics including principal component analysis. Heatmap, Column scatter graph, Violin plot and Volcano plot were generated using GraphPad prism software ver. 7 (GraphPad Software Inc., San Diego, CA, USA). Binary logistic regression for biomarker analysis was conducted using IBM SPSS statistics for Windows, version 25.0 (IBM Corp.). Receiver operating characteristic (ROC) analysis was generated using MedCalc version 12.7.0.0 (MedCalc Software, Ostend, Belgium) and evaluated with permutation test by MetaboAnalyst 4.0. Co‐inertia analysis was performed in the M2IA server (http://m2ia.met‐bioinformatics.cn/).^23^ Interomic correlation matrix between individual metabolite and microbial composition (at genus level) was constructed based on Spearman rank analysis (package stats in the software R).

### 2.6 Additional methods

Additional methods are provided in Supporting Information Methods online.

## 3 RESULTS

### 3.1 *L. lactis* and *P. pentosaceus* suppress the progression of NAFLD by modulating gut‐liver axis

**FIGURE 1:**
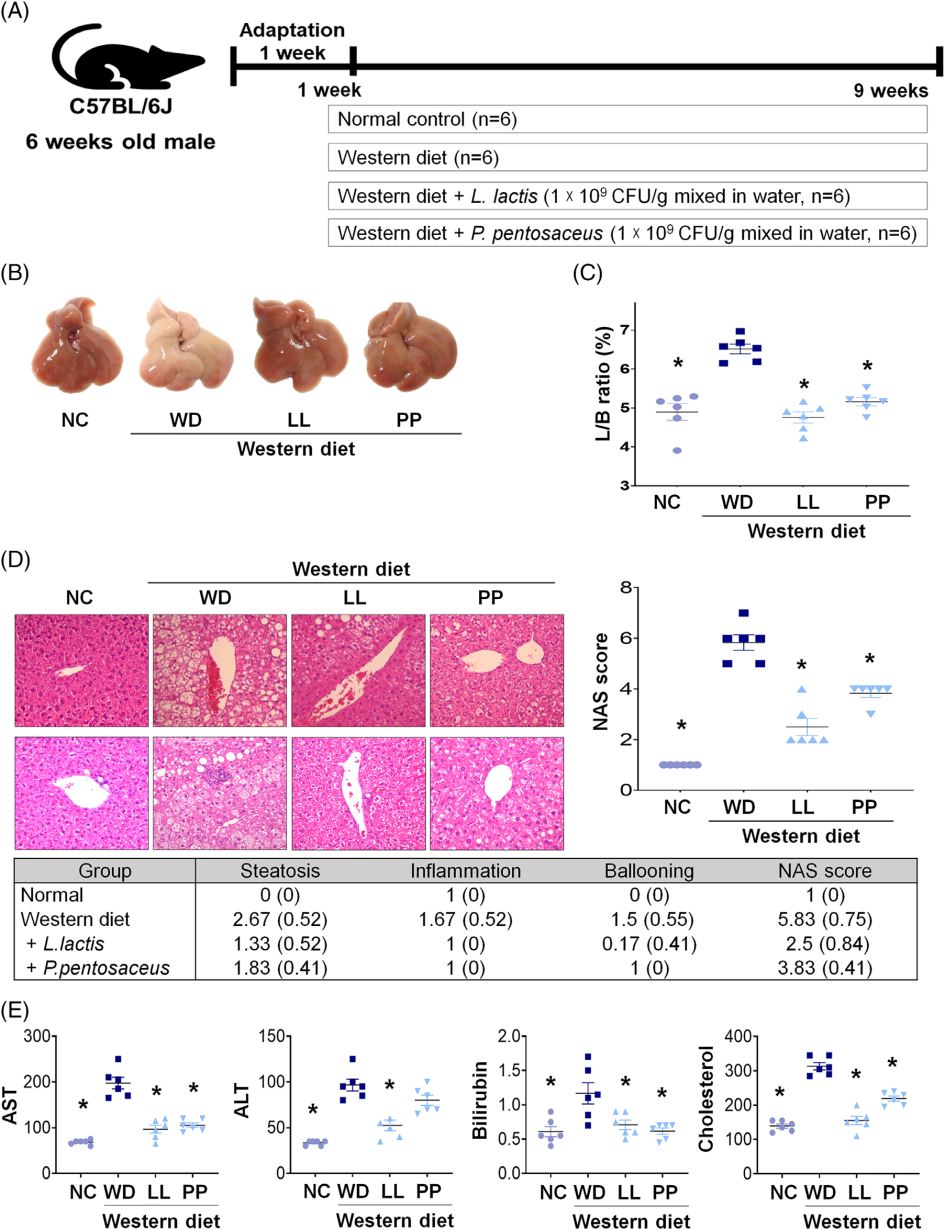
**Effect of *Lactobacillus* and *Pediococcus* on the Western diet‐induced liver disease**. (A) Experiment design of WD model. (B) Gross specimen of mice liver. (C) L/B ratio. (D) Pathological effects of strains on the liver. Hematoxylin and eosin staining of liver sections were analyzed (x 20) and analyze to NAFLD activity score (NAS). (E) Liver function test and cholesterol level of mice. *p < 0.05 as compared with WD. L/B, Liver weight/body weight; LL, *Lactobacillus lactis*; NC, normal control; PP, *Pediococcus pentosaceus;* WD, Western diet

Mice were divided into four groups (*n* = 6 per group): normal diet (NC), WD and WD supplemented with *L. lactis* (LL), and *P. pentosaceus* (PP) groups (Figure 1A). The *L. lactis* and *P. pentosaceus* supplementations significantly improved liver/body weight ratio (%) when compared with that of the WD group (Figure 1B,C) (*p *< 0.001). In the liver pathology, WD‐induced steatosis and inflammation were reduced in the two strain groups. NAFLD activity score (NAS) was improved in the two strain groups. The strain groups (LL and PP) showed complete remission with a score of 1 in the inflammation grade (Figure 1D). The mean levels of AST, ALT, bilirubin and cholesterol were significantly reduced by the strain supplementation (Figure 1E).

**FIGURE 2:**
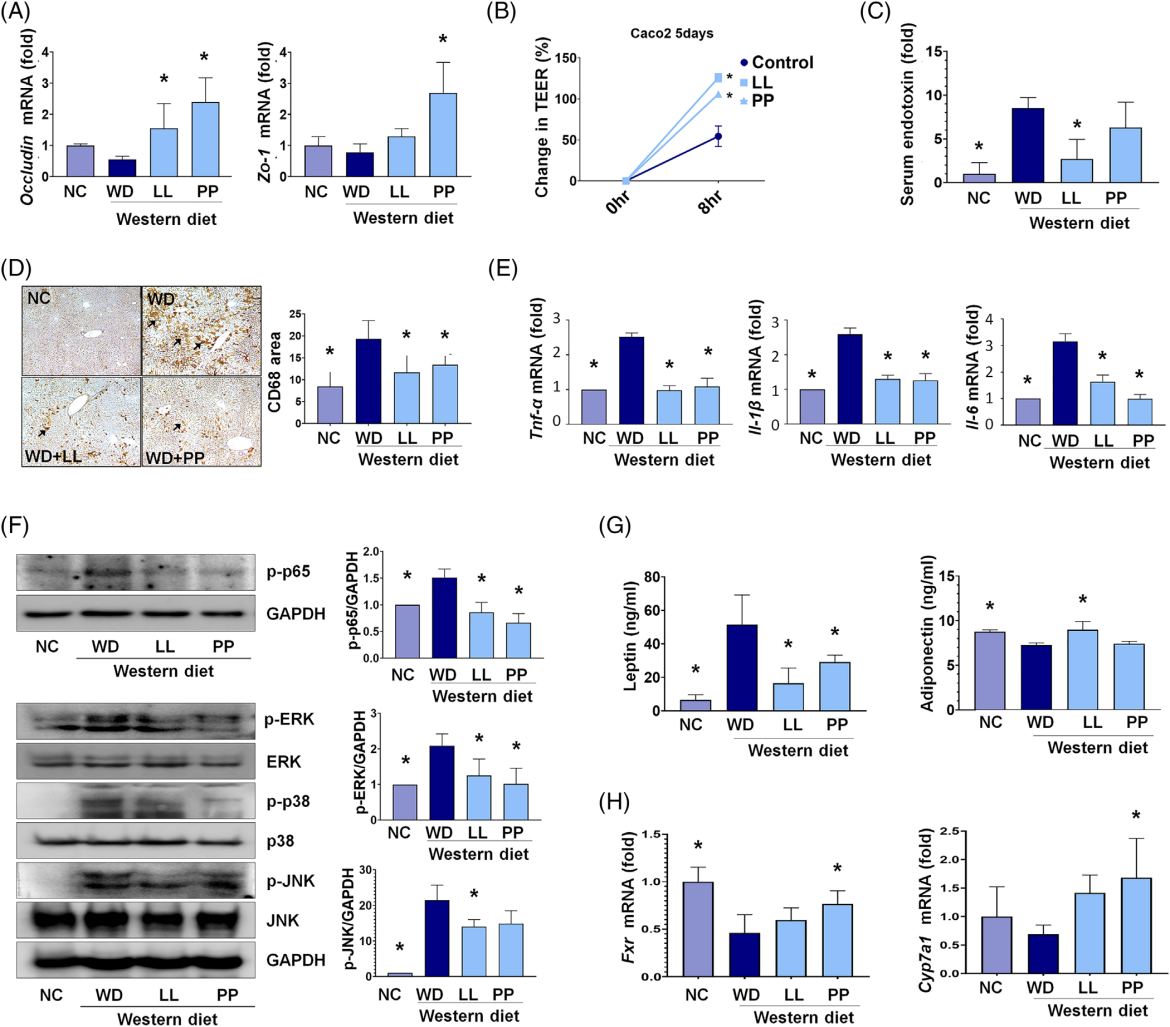
**Effects of *L. lactis* and *P. pentosaceus* on gut‐liver axis**. (A) The expression of tight junction markers, occludin and Zo‐1, in the mice intestine (*n* = 5/group). (B) Trans‐epithelial electrical resistance assay using Caco2 cell. (C) The levels of endotoxin in mice serum (*n* = 5/group) using LAL assay kit. (D) Representative microphotographs and measured areas of CD68 immunohistochemistry. (E) The levels of inflammatory cytokines tumor necrosis factor (TNF)‐α, interleukin (IL)‐1β and interleukin (IL)‐6 in mice liver (*n* = 5/group). (F) Effects of strains on WD‐induced activation of MAPKs and NF‐kB in mice liver. (G) The analysis of adipokines such as leptin and adiponectin in mice liver (*n* = 5/group). (H) The levels of bile acid (BA) regulation‐related genes, Cyp7A1 and FXR, in mice liver (*n* = 5/group). *p < 0.05 as compared with WD. LL, *Lactobacillus lactis*; MAPKs, Mitogen‐activated protein kinases; NC, normal control; NF‐kB Nuclear factor kappa‐light‐chain‐enhancer of activated B cells; PP, *Pediococcus pentosaceus;* WD, Western diet

In the gut‐tight junction, the LL and PP groups showed the overexpression of occludin and ZO‐1 genes compared with the ones in the other groups (Figure 2A). Besides, the treatment of LL and PP on Caco‐2 cells increased the trans‐epithelial electrical resistance values by 2.3‐ and 1.9‐fold, respectively, compared with those of non‐treated controls (Figure 2B). The elevated levels of serum endotoxin by WD were significantly reduced in the LL group (Figure 2C). These results suggest that LL and PP supplementations improved intestinal‐barrier function and reduced bloodstream endotoxin infiltration from the intestine.

Immunohistochemical analyses of hepatic CD68, a marker for macrophages showed the significant reduction of the stained areas in the LL and PP groups compared to the WD group (Figure 2D). In the liver tissue, elevated inflammatory cytokines including tumor necrosis factor (TNF)‐α, interleukin (IL)‐1β and IL‐6 were substantially normalized in the LL and PP groups (Figure 2E, Figures S2D and S3A). In addition, Mitogen‐activated protein kinases (MAPKs) (p38, c‐Jun NH2‐terminal kinase) and extracellular signal‐regulated kinase, which participate in the expression of pro‐inflammatory mediators, were down‐regulated in the LL and PP groups (Figure 2F). Likewise, pro‐inflammatory adipokines including retinol‐binding protein 4 and leptin showed decreased levels in the LL and PP groups (Figure S3B). On the contrary, anti‐inflammatory adipokine, adiponectin was significantly elevated in the LL group compared with that in the WD group. Bile acid‐related genes, Cyp7A1 and farnesoid X receptor (FXR) were partly normalized by the LL and PP supplementations (Figure 2G,H). Lipid metabolism‐associated genes were differentially regulated by the supplementation of two strains (Figure S3C).

### 3.2 The microbial taxonomic and metabolic profiles are altered in the WD group

**FIGURE 3:**
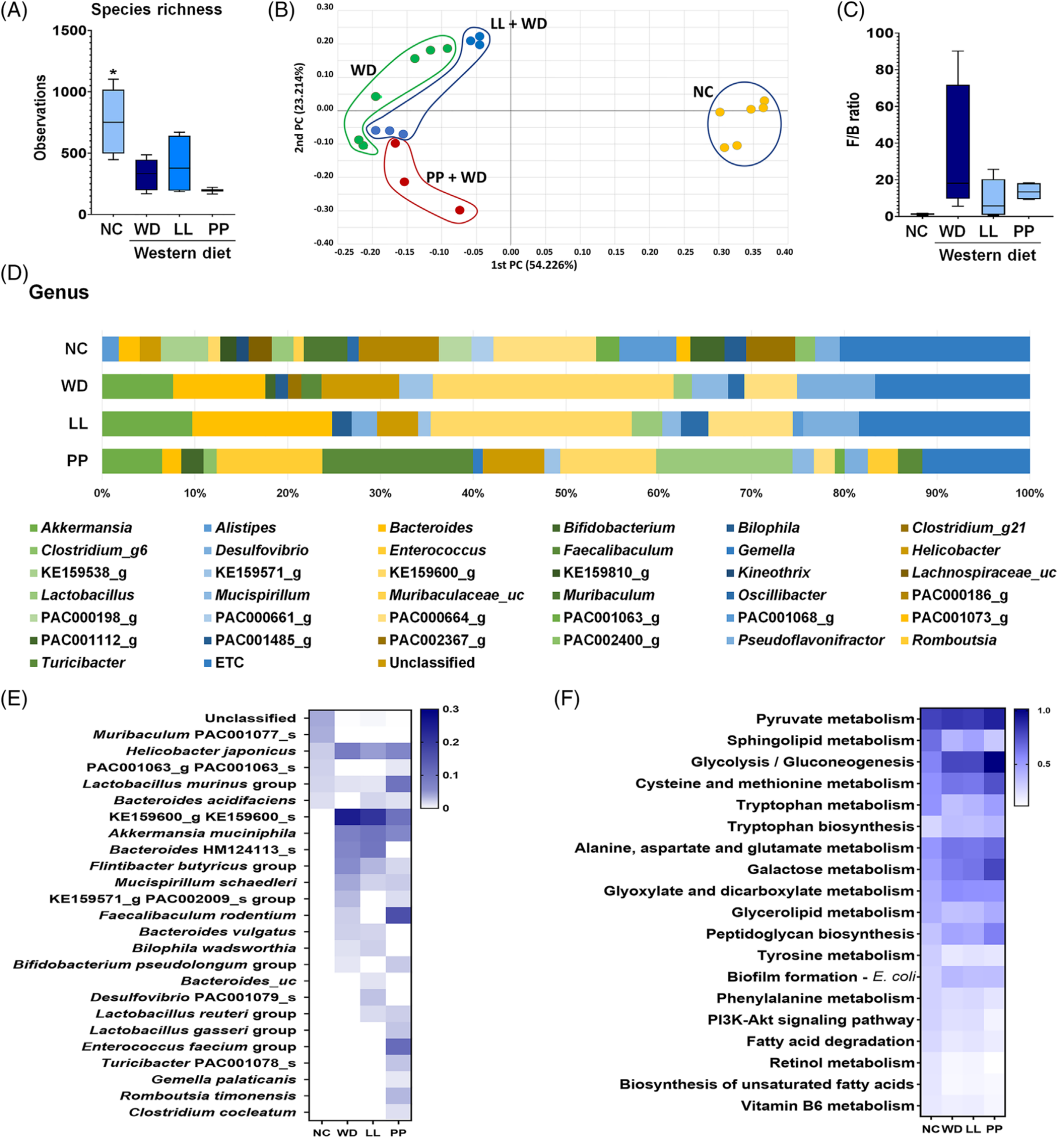
**The alteration in the microbial taxonomic composition of the Western diet‐mice caecal samples**. (A) The alpha diversity based on species richness. (B) The beta diversity by principal coordinate analysis (Bray‐Curtis distance), and (C) *Firmicutes* to *Bacteroidetes* (F/B) ratio as compared among four different groups (NC, WD, LL, and PP). (D) Relative abundances of caecal microbiome at genus level in different experimental groups. (E) Heatmap analysis for significantly different species. (F) Comparative analysis of the estimated functional profiles based on KEGG orthology in different experimental group. KEGG, Kyoto encyclopedia of genes and genomes LL, *Lactobacillus lactis*; NC, normal control; PP, *Pediococcus pentosaceus;* WD, Western diet

We analyzed the profiles of 16S rRNA gene amplicon sequencing and compared the compositional characteristics according to the different treatment. The lower species richness was determined in all the WD‐treated groups (WD, LL and PP) relative to the normal control (NC) group, but it was not statistically significant (Figure 3A). The microbial taxonomic profiles indicated the clear discrimination between the NC and all other groups, in which the LL and PP groups were partially separated from the WD group based on principal coordination analysis (β‐diversity, PCoA based on Bray‐Curtis matrix) (Figure 3B). The *Firmicutes*‐to‐*Bacteroidetes* ratio (F/B ratio) was marginally decreased in the LL and PP groups to the level of the NC group (Figure 3C). Overall microbial compositions at phylum level were changed by the supplementation of two strains (Figure S3D). At the genus level, each group presented different compositions (Figure 3D). The genera, *Helicobacter*, KE159600_g, *Mucispirillum, Pseudoflavonifractor, Clostridium*_g21 and *Faecalibaculum* were enriched in the WD group compared to the NC, LL and PP groups. On the contrary, *Lactobacillus* and PAC000664_g were depleted in the WD group (Figure 3D). Heatmap analysis showed the compositional characteristic among the genus that were significantly different according to each group, including *Bacteroides* HM124113_s *Flintibacter butyricus* group, *Mucispirillum schaedleri*, *Faecalibaculum rodentium* and *Bacteroides vulgatus* (Figure 3E). The functional profiles of the gut microbial communities inferred the potential role of tryptophan metabolism, which was similarly regulated among the NC, LL and PP groups compared to the WD group (Figure 3F). The comparative abundance of microbial genes related to selected metabolic pathways and signaling pathways is presented in Table S2.

### 3.3 The caecal metabolome is globally dys‐regulated by the WD

**FIGURE 4:**
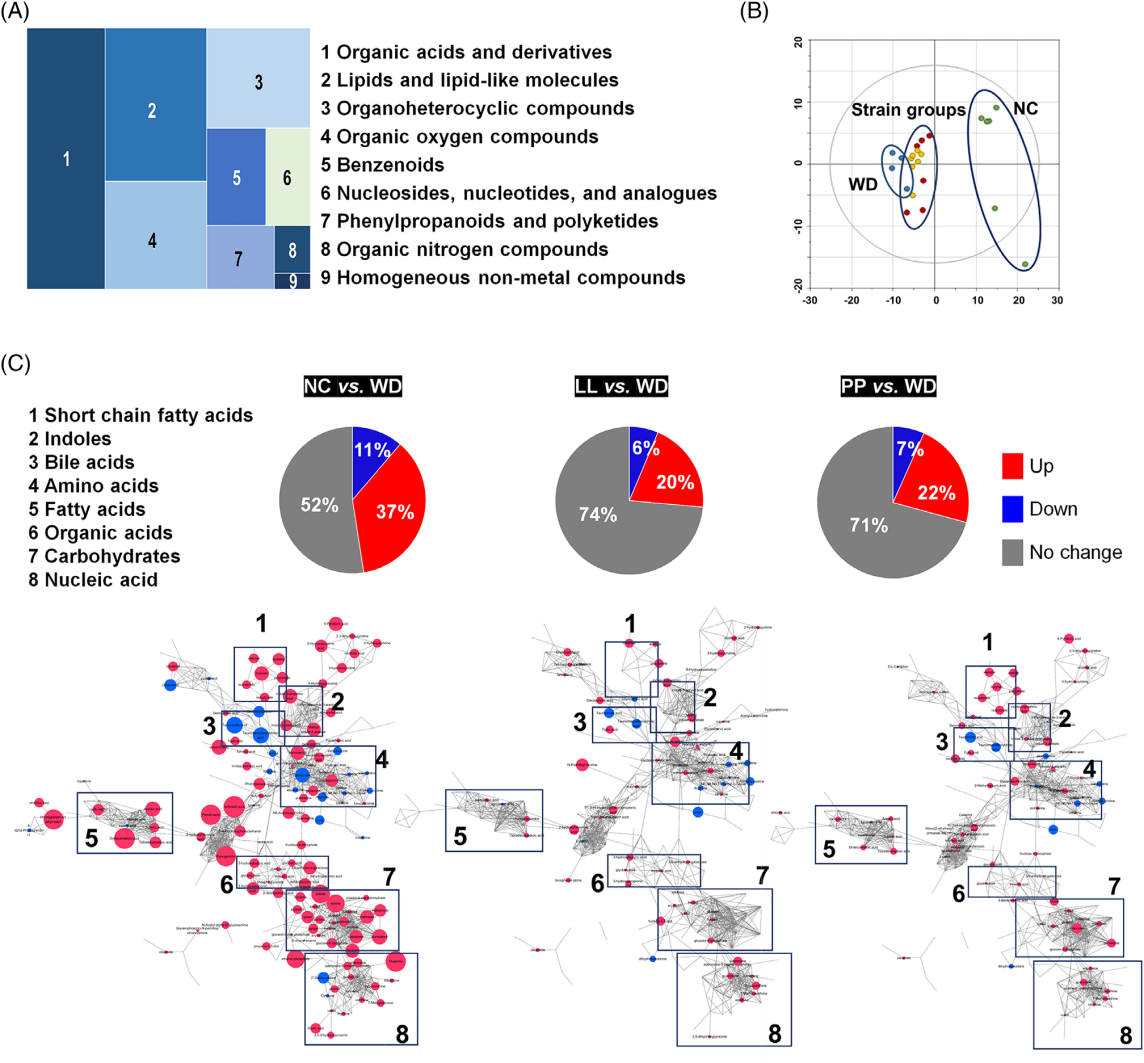
**The caecal metabolomic dysregulation by Western diet and the normalization by the supplementation with *L. lactis* and *P. pentosaceus***. (A) Chemical classification of identified metabolites in mouse caecum provided by HMDB (http://www.hmdb.ca). A total of 256 compounds (91%) are categorized into nine super classes. (B) The score scatter plot of 282 caecal metabolites by principal component analysis (PCA). Most variation was imposed by PC1 (30.8%) and PC2 (12.3%). *p < 0.05 as compared with WD. (C) Overview of the metabolic features. Pie charts present the number of metabolites that were significantly different in other groups, respectively compared to WD (Student's *t*‐test, *p *< 0.05). Red and blue colours present significantly higher or lower abundance in other groups, respectively compared to WD (*p *< 0.05). The network is constructed based on chemical structural similarity (Tanimoto score) and KEGG reaction pair (substrate‐product relation), which results in distinctive metabolic modules indicated by box. Red and blue colours present significantly higher or lower abundant in NC, LL, and PP groups, respectively compared to WD (Student's t test, *p* < 0.05). Node sizes are determined by the ratios. HMDB, human metabolome database; KEGG, Kyoto encyclopedia of genes and genomes; LL, *Lactobacillus lactis*; NC, normal control; PC1, Climatic index 1; PC2, Climatic index 2; PP, *Pediococcus pentosaceus;* WD, Western diet

To comprehensively characterize caecal metabolic changes, we performed untargeted and targeted metabolite profiling based on GC‐ and LC‐MS analysis. Metabolic signals were assigned to 282 unique compounds based on reference comparisons, spectra library searching and retention time indexes. The chemical ontology analysis classified the compounds, and approximately 50% of metabolites were categorized as organic acids and lipid molecules (Figure 4A). The sub‐categories of the major classes were as follows: carboxylic acids, fatty acyls and steroids accounted for 60, 32 and 14 compounds, respectively (Table S3). Principal component analysis showed the distinctive clusters between the NC group and the others, which was similar with the taxonomic profiles (Figure 4B).

We, then, determined caecal metabolite contents that were shifted by the WD. The significant differences were found in 135 compounds (Table S4). Eleven per cent of metabolites were significantly enriched in the WD group, whereas 37 per cent were depleted when compared to those in the NC group. The highest depletion was determined in an indole compound, 5‐hydroxyindole‐3‐acetic acid of the WD group (Table S4). Other indole derivatives were concomitantly depleted, including indole‐3‐propionic acid, methyl indole‐3‐acetic acid, indole‐3‐acetic acid and indole‐3‐acrylic acid. In addition, the significant down‐regulation was identified in the metabolites associated with carbohydrate metabolism based on pathway overrepresentation analysis (Figure S4A). All monosaccharides were significantly depleted in the WD group compared to the NC group. Among the intestinal monosaccharides, glucose and xylose were significantly depleted in the WD group, respectively compared to those in the other groups (Figure S5, Table S5). The strain‐fed groups showed glucose levels equivalent to those in the NC group. Fructose was not differentially regulated among the WD groups, whereas galactose and mannose levels in the PP group were at comparable levels to those in the NC group (Figure S5).

On the contrary, taurine conjugated BAs were the compounds with the highest increases in WD group compared to NC group. Taurocholic acid and taurochenodeoxycholic acid were found 50‐/43‐fold increases in WD group compared to NC group, respectively. Others were glutamic acid, cholesterol, 2′‐deoxycytidine and glycocholic acid (>10‐fold changes). Overall, the metabolites with the highest enrichment were associated with amino acid metabolism and primary BA biosynthesis. (Figure S4B).

### 3.4 Potential protective therapeutics are identified from common metabolic signatures among NC and two strain‐fed groups

**FIGURE 5:**
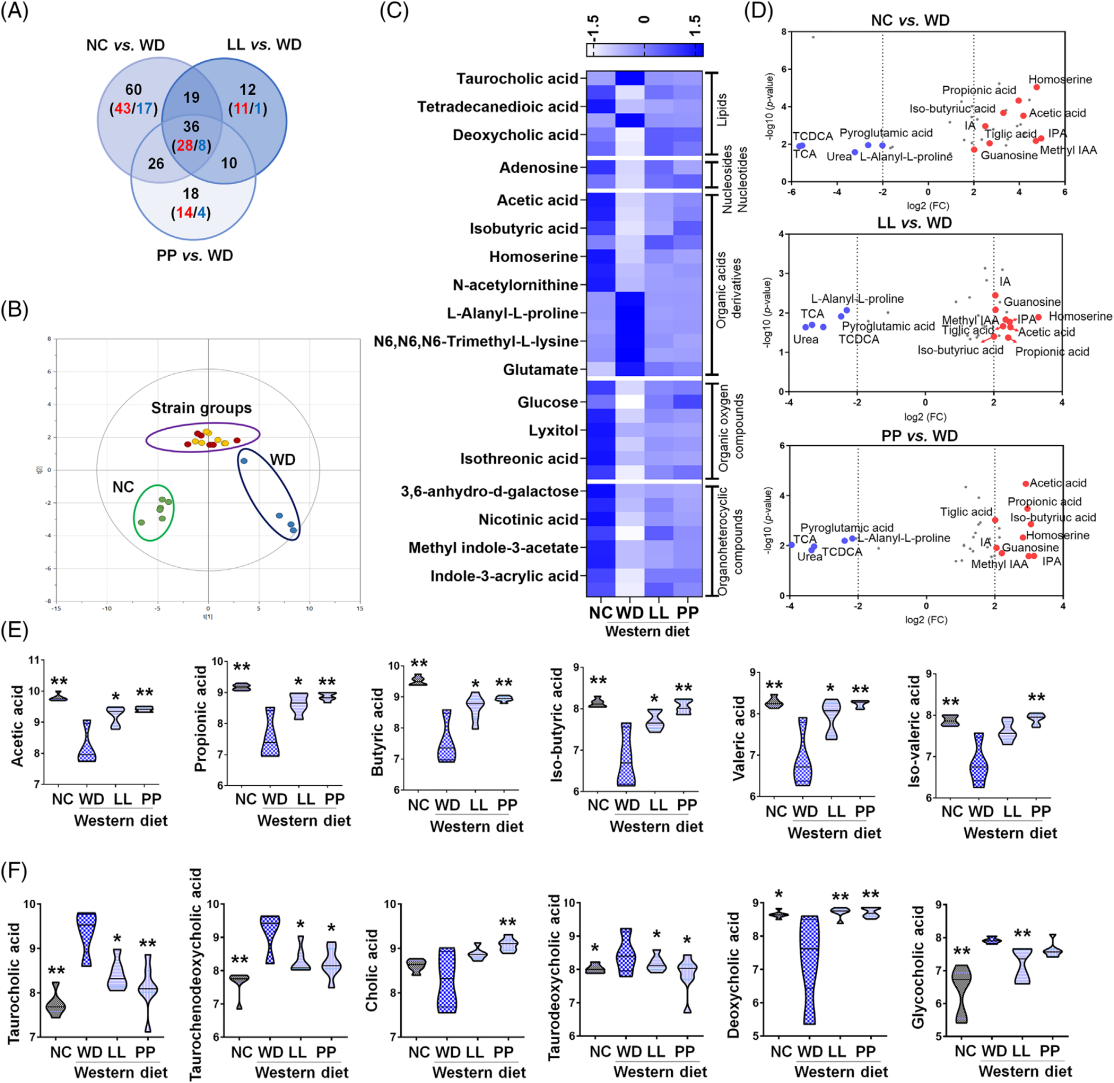
**The characteristic alteration in the major caecal metabolites according to different diet types and strains supplementation**. (A) The metabolites that show common abundance pattern in other groups compared to WD group. A total of 36 metabolites are significantly different in all three groups. (B) The score scatter plot of the 36 metabolites by principal component analysis. (C) Heatmap of auto‐scaled abundances (mean‐centered and divided by the standard deviation of each variable) of the common metabolites. (D) Volcano plot of the common metabolites (36 metabolites) that are significantly different in all three groups, respectively compared to WD group. The x‐axis is log2‐fold change, and the y‐axis is log10‐*p* value. Indole derivatives (indole‐3‐propionic acid and methyl indole‐3‐acetic acid) show the highest fold‐increases in all three groups. Bile acids (taurocholic acid and taurochenodeoxycholic acid) present the highest fold‐decreases in all three groups. The name of metabolites in common among three groups is only visualized. The levels of caecal short chain fatty acids (SCFAs) (E) and bile acids (BAs) (F) in different diet groups (*n* = 4–7). The log10‐transformed abundances (ion intensities) are shown as violin plot. *p < 0.05 as compared with WD group by Mann–Whitney *U* test. **p < 0.05 as compared with WD group by nonparametric Kruskal–Wallis test and Dunn's test adjusted by Benjamini‐Hochberg correction. LL, *Lactobacillus lactis*; NC, normal control; PP, *Pediococcus pentosaceus;* WD, Western diet

We explored common metabolic features among the NC, LL and PP groups relative to the WD group, which may screen keystone metabolites effectively and determine the underlying molecular mechanistic in alleviating the NAFLD progression. Accordingly, we constructed an integrated metabolic network (MetaMapp) that was tiered by chemical structural similarity (*Tanimoto* score) and enzymatic reaction connectivity (KEGG reaction pair). The network provided a general overview at the level of the metabolic module and comprehensive details at the individual metabolite level.^24^ Overall, the LL and PP groups showed compatible patterns in major metabolic modules, which coincided with the similar levels of the preventive effect of WD‐induced liver damage (Figure 4C). We further interrogated the common metabolites that were similarly regulated in the NC and the strain‐fed groups. A total of 36 metabolites showed similar patterns among the three groups compared to the WD group (Figure 5A, Figure S6). The metabolic profiles of the 36 common metabolites were clearly discriminated among the three groups, the NC group, two strain‐fed groups (LL and PP) and the WD group (Figure 5B).

Particularly, the common metabolites were classified into three main entities: SCFAs, BAs and tryptophan metabolites. The WD group was featured by the significant decreases in the SCFAs except iso‐valeric acid compared to the other three groups (*p *< 0.05) (Figure 5C,D). The LL group showed marginal differences in SCFAs after multiple‐comparison adjustment whereas the PP group presented the significant differences in all SCFAs compared to the WD group (Figure 5E). The differences were not significant between the two strain‐fed groups (Table S6). On the contrary, primary BAs conjugated with taurine were most dramatically up‐regulated in the WD group, compared to those in the other groups (Figure 5D,F). Glycocholic acid was significantly enriched in the WD group relative to that in the NC and LL groups (Figure 5D,F). Secondary BAs following deconjugation/dehydroxylation of primary BAs presented a decreasing tendency in the WD group except taurodeoxycholic acid (Table S6).

**FIGURE 6:**
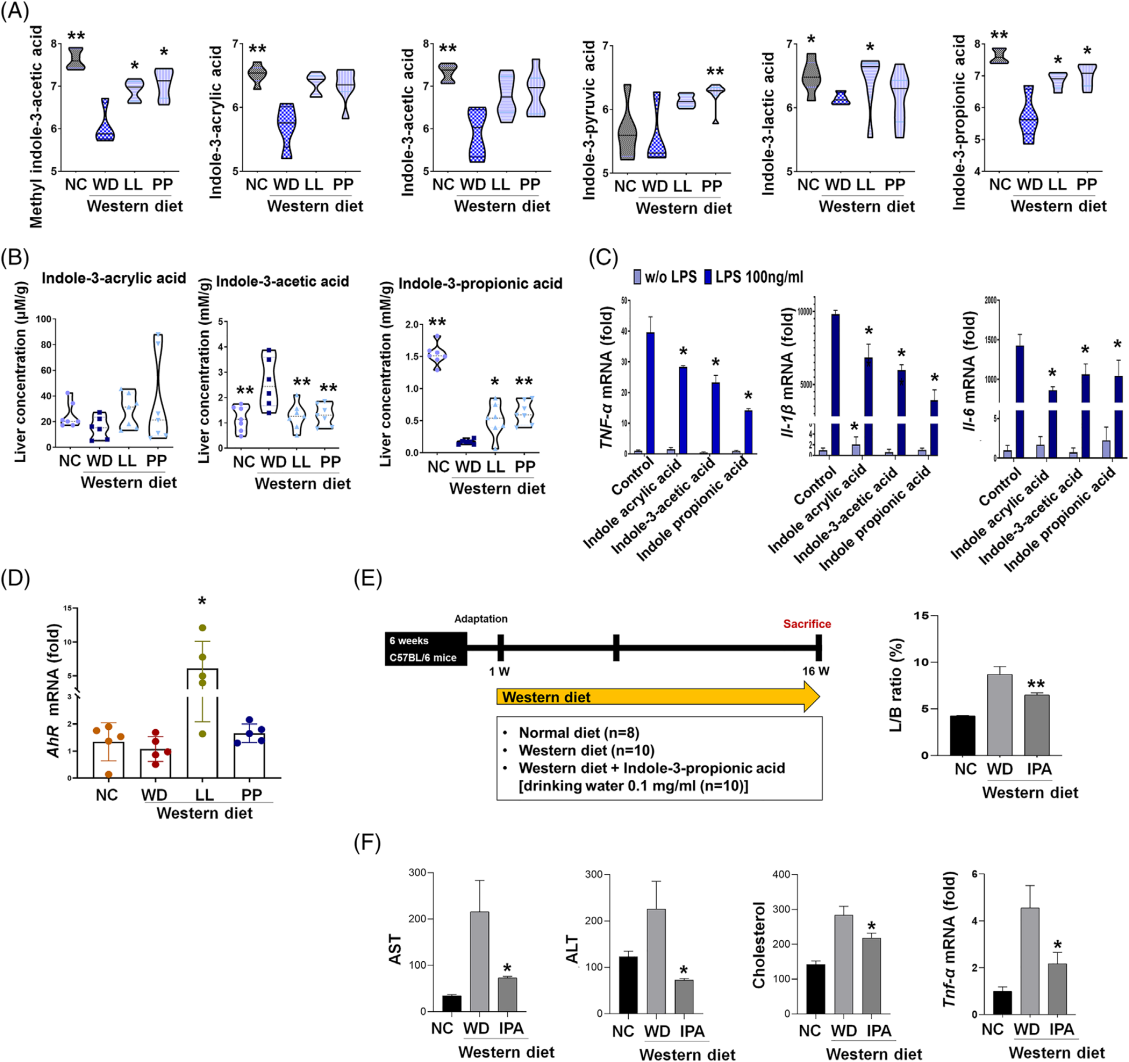
**Ameliorative effects of gut microbe‐derived indoles on non‐alcoholic fatty liver disease (NAFLD) progression**. The levels of indoles in caecal samples (A) and livers (B) in different diet groups (*n* = 4‐7). The log10‐transformed abundances (ion intensities) are shown as violin plot. *p < 0.05 as compared with WD group by Mann–Whitney *U* test. **p < 0.05 as compared with WD group by nonparametric Kruskal–Wallis test and Dunn's test adjusted by Benjamini–Hochberg correction. (C) Anti‐inflammatory effects of indoles on Raw 264.7 cell exposed to LPS (100 ng/ml, *n* = 5). Following the subsequent indole‐treatment (indole acrylic acid, 100 μM; indole‐3‐acetic acid, 500 μM; indole‐propionic acid, 100 μM), inflammatory cytokine gene expression is analyzed based on qRT‐PCR. *p < 0.05. (D) The expression levels of aryl hydrocarbon receptor. (E) Experiment design of WD indole mice model and L/B ratio (F) level of liver enzymes, cholesterol, and cytokine. IPA, indole‐propionic acid; L/B, Liver weight/body weight; LPS, lipopolysaccharides; LL, *Lactobacillus lactis*; NC, normal control; PP, *Pediococcus pentosaceus*; qRT‐PCR, quantitative reverse transcription polymerase chain reaction; WD, Western diet

Among tryptophan metabolites, indole‐3‐propionic acid and methyl indole‐3‐acetic acid showed the most consistent and significant difference in the NC and the strain‐fed groups (LL and PP) compared to WD (Figures 5D and 6A). Indole‐3‐acrylic acid and indole‐3‐acetic acid in LL and PP were found marginally different from the ones of WD (Figures 5D and 6A, Table S7). Indole‐3‐lactic acid was at normal level in LL, whereas indole‐3‐pyruvic acid was specifically higher in PP. The subsequent analysis of serum indoles showed compatible patterns to the caecal indoles (Figure S7). Indole‐3‐acrylic acid and indole‐3‐acetic acid were significantly higher in NC and LL groups compared to WD, while indole‐3‐propionic acid was not altered in both strain‐fed groups. Of particular, hepatic indole‐3‐propionic acid showed the significant enrichment in three groups (NC, LL and PP) compared to WD (Figure 6B), mirroring the caecal contents. Indole‐3‐acrylic acid was marginally higher in NC, LL and PP than in WD, whereas indole‐3‐acetic acids were substantially lower in the three groups compared to WD, contrast to the pattern in caecum and serum.

Indole derivatives are the ligands for aryl hydrocarbon receptor (AHR), which is implicated in intestinal barrier function and immune system (e.g., anti‐inflammation) by mediating and integrating various environmental cues.^25^, ^26^ In our result, the decreased expression of AHR was restored in the LL group (Figure 6D). For the evaluation of anti‐inflammatory effect of tryptophan metabolites, we measured pro‐inflammatory cytokines in lipopolysaccharides (LPS)‐stimulated macrophages. Raw264.7 cells were treated indole‐3‐acetic acid (100 μM), indole‐3‐propionic acid (100 μM)^27^ or indole‐3‐acrylic acid (500 μM)^28^ following LPS treatment. Indole compounds significantly decreased the expression of TNF‐α, IL‐1β and IL‐6 mRNA (Figure 6C). The result was further validated in the animal study, in which indole‐3‐propionic acid normalized liver weight/body weight (L/B) ratio, liver enzyme, cholesterol level and cytokine level (Figures 6E,F).

### 3.5 The gut metabolic profiles of human subjects mirror the ones of NAFLD mice model

**FIGURE 7:**
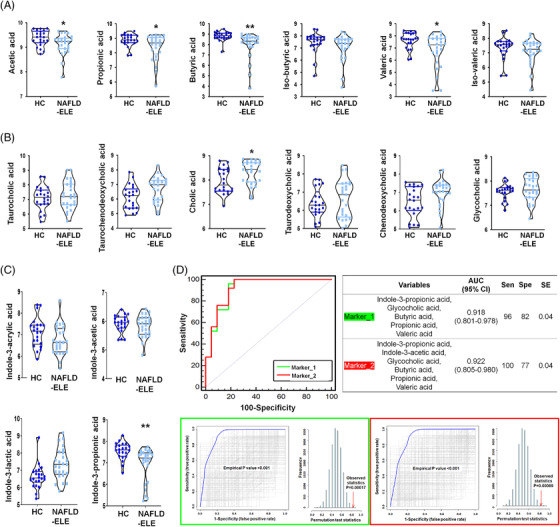
**Human faecal metabolomic features in the non‐alcoholic fatty liver disease (NAFLD) patients**. The levels of human faecal short chain fatty acids (SCFAs) (A), bile acids (BAs) (B), and indoles (C) in two different groups, healthy control (HC) (*n* = 22) and the patients with NAFLD‐elevated liver enzyme (ELE) (*n* = 25). The log10‐transformed abundances (ion intensities) are shown as violin plot. *p < 0.05 as compared with HC group based on Student's *t*‐test. **p < 0.05 as compared with HC after multiple comparison adjustment by false discovery rate. (D) Receiver operating characteristic (ROC) curve analysis of biomarker panel included by multiple faecal metabolites for discriminating the HC (*n* = 22) and non‐alcoholic steatohepatitis (NASH) (*n* = 25). Each biomarker cluster includes Marker_1 (indole‐3‐propionic acid, glycocholic acid, propionic acid, butyric acid, valeric acid), Marker_2 (indole‐3‐propionic acid, indole‐3‐acetic acid, glycocholic acid, propionic acid, butyric acid, valeric acid). The area under curve value is 0.918 (95% confidence interval: 0.801–0.978) and 0.922 (95% confidence interval: 0.805–0.980), respectively. Optimal cutoff is determined using the closest to top‐left corner, and the 95% confidence interval is calculated using 1000‐bootstrapping. The area under ROC curve and predicative accuracy are calculated through 1000 times permutation test

Parallel to the mouse model, the levels of the main SCFAs were observed at significantly lower levels in the patients with NAFLD‐elevated liver enzyme (NAFLD‐ELE) than healthy controls (HCs) (*p *< 0.05) (Figure 7A and Table S6). On the contrary, the faecal BAs showed elevated levels in the patients diagnosed with NAFLD‐ELE (Figure 7B), which were not identical to the profiles of the caecal BAs in the WD group. Nonetheless, comparable dysregulation was identified for faecal taurocholic acid (*p *= 0.122, false discovery rate (FDR) = 0.256) and glycocholic acid (*p *= 0.213, FDR = 0.298) in the patients with NAFLD‐ELE. Indole‐3‐propionic acid was at significantly‐reduced level in the patient group (Student's *t*‐test, *p *< 0.01). Indole‐3‐acrylic acid and indole‐3‐acetic acid were marginally reduced (*p *= 0.656, 0.454), whereas indole‐3‐lactic acid was moderately increased in the NAFLD patients compared to HC (Student's *t*‐test, *p *= 0.180) (Figure 7C). We performed binary logistic regression analysis to examine whether a composed set of stool metabolites can predict NAFLD‐ELE (*n* = 25) against HC (*n* = 22). Based on the key metabolites (SCFAs, indoles and BAs), we applied ROC curve analysis for accuracy, specificity and sensitivity. The area under the curve (AUC) for a linear composite of five stool metabolites (indole‐3‐propionic acid, glycocholic acid, propionic acid, butyric acid, valeric acid) was 0.918 (95% CI, 0.801–0.978) for the NAFLD‐ELE against the HCs with 0.96 of sensitivity and 0.818 of specificity (Figure 7D). Also, a linear composite of six metabolites (indole‐3‐propionic acid, indole‐3‐acetic acid, glycocholic acid, propionic acid, butyric acid, valeric acid) showed excellent discrimination power (AUC [95% CI]: 0.922 [0.805–0.980]). The accuracy of the biomarker panels was evaluated based on a permutation test (1000‐fold).

### 3.6 Microbial community changes according to the progression of NAFLD in human subjects

**FIGURE 8:**
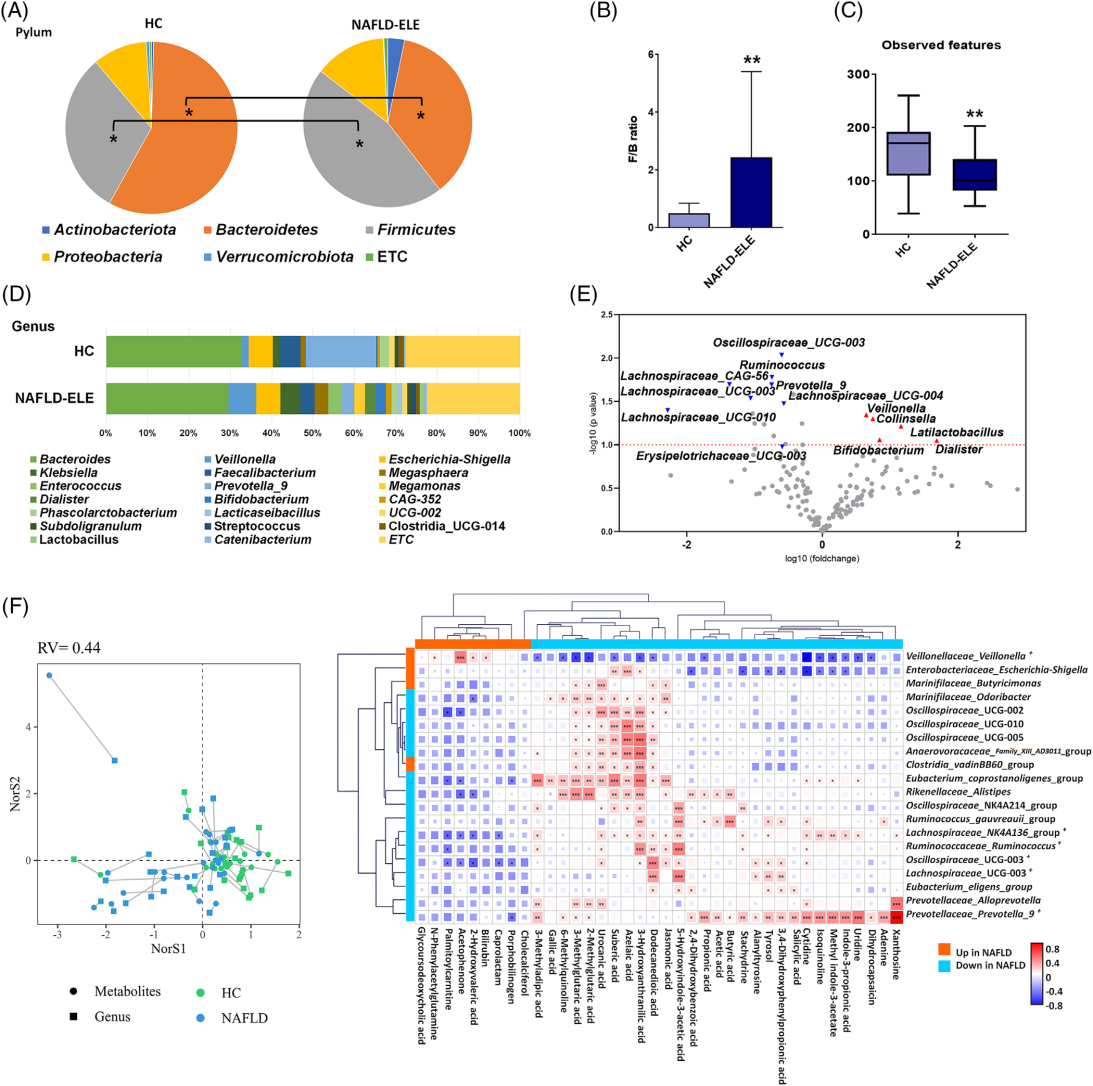
**Microbial community profiles of human in response to different diet type and microbial preventive therapy**. (A) Pie charts of phylum distribution in human stool. The distribution of phylum was compared between healthy control (HC) (*n* = 22) control and non‐alcoholic fatty liver disease‐elevated liver enzyme (NAFLD‐ELE) (*n* = 25). *p < 0.05 (B) *Firmicutes* to *Bacteroidetes* ratio. (C) Box and whisker plot of Alpha‐diversity indices. *p < 0.05 and **p < 0.01 as compared between the NC and the NAFLD‐ELE groups based on Mann–Whitney *U* test. (D) Relative abundance of faecal microbiome at the genus level in HC and NAFLD‐ELE. (E) Volcano plot of stool microorganisms at genus level. The x‐axis presents log10 transformed fold‐change, and y‐axis presents ‐log10 transformed *p*‐value calculated by Student's *t*‐test. Red and blue colours present higher or lower abundance in NAFLD‐ELE compared to HC. (F) Co‐inertia analysis of metabolomic and taxonomic profiles. The x‐ and y‐axis present the first two vectors that most explain the variance composed with integrative metabolomic and taxonomic profiles. Circles and squares represent the faecal metabolome and microbiome, respectively. Green and blue‐sky colours indicate NC and NAFLD groups, respectively. Lines connect the faecal metabolome and microbiome from the same individual. The shorter the length of line is, the stronger the level of association is. (G) Co‐occurrence matrix of individual metabolite and microbial composition. The correlation structure consists of gut microbial feature (genus level) and the metabolic features that showed the significant alteration in the NAFLD patients. The correlation coefficient was calculated based on Spearman rank analysis. *p < 0.05, **p < 0.01, and ***p < 0.001

We comparatively analyzed the gut microbial composition based on 16S rRNA gene amplicon sequencing data from human stool. The composition of phyla was significantly different according to the progression of liver diseases. The *Firmicutes* were enriched in the NAFLE‐ELE group, while *Bacteroidetes* were more abundant in the NC group (Figure 8A). Accordingly, the F/B ratio in the NAFLD‐ELE was significantly higher than that of the NC group (Figure 8B) (*p *< 0.01). In the alpha diversity analysis, the NAFLD‐ELE group had significantly reduced microbial richness relative to the NC group (Figure 8C). At the genus level, *Bacteroides*, *Prevotella_9* and *Faecalibacterium* dominated the faecal microbiome in the NC group. *Bacteroides*, *Veillonella* and *Klebsiella* showed the compositional dominance in the NAFLD‐ELE group (Figure 8D). The genera, *Veillonella*, *Collinsella*, *Latilactobacillus*, *Dialister* and *Bifidobacterium* were enriched in the NAFLD‐ELE group compared to the NC group. On the contrary, *Oscillospiraceae_UCG‐003*, *Ruminococcus*, *prevotella‐9*, *Lachnospiraceae_UCG‐004*, *Lachnospiraceae_UCG‐003*, *Lachnospiraceae_UCG‐010* and *Erysipelotrichaceae_UCG‐003* were depleted in the NAFLD‐ELE group (Figure 8E).

### 3.7 Integrative profiles of faecal metabolome and microbiome underpin the echo‐systemic dysbiosis in the NAFLD patients

The global similarity was evaluated by co‐inertia analysis, in which the short distance between the metabolome and microbiome implied the strong association. The inter‐omic data matrix led to the clear discrimination between HC and NAFLD‐ELE groups mainly along the first axis (NorS1) (Figure 8F). The inter‐omic association was further characterized at the levels of individual metabolite and genus based on Spearman correlation. The correlation matrix was particularized based on the metabolites with the significant alteration in the NAFLD patients and top 20 genera with the highest degree (Figure 8G). The *Oscillospiraceae* family showed the common feature, the significant positive correlation with urocanic acid and 3‐hydroxyanthranilic acid. Likewise, the family was strongly associated with azelaic acid and suberic acid except the genus, *Oscillospiraceae_UCG‐003*. The genera, *Prevotella‐9*, *Coprostanoligenes_group*, *Alistipes* and *Lachnospiraceae_NK4A136_group* showed the highest connectivity with metabolites. In particular, *Prevotella‐9* presented the co‐occurrence with key gut metabolites including SCFAs (acetic acid, propionic acid, and butyric acid) and indole‐3‐propionic acid. Among metabolites, 3‐hydroxyanthranilic acid, 5‐hydroxyindole‐3‐acetic acid, dodecanedioic acid and urocanic acid were highly associated with gut microbiome (Figure 8G).

## 4 DISCUSSION

Probiotics supplementation, including *L. lactis* and *P. pentosaceus*, is implicated with beneficial effects in various diseases such as enteritis, inflammation and hypertension.^29^, ^30^ Growing body of evidence suggests that gut‐liver axis plays an important role in the pathophysiology of NAFLD, and that probiotics can modulate the elements of gut‐liver axis, especially gut microbiota.^7^, ^31^, ^32^ Nonetheless, the comprehensive mechanistic remains still elusive. In this regard, our current investigation is the first report on the accomplishment of multiomics‐based mechanistic elucidation of LL‐ and PP‐supplementation therapy for NAFLD progression.

First, our current study recapitulated the potential of probiotics‐based therapy for NAFLD. LL and PP supplementation significantly improved the liver function, including NAS score, and serum chemistry markers (AST, ALT, bilirubin, and total cholesterol) as compared to the WD group. The improvement was coupled to the recovery of histological signature and phenotypes (body weight and L/B ratio). The preventive remedies decreased serum endotoxin level by restoring tight junction and curved release of pro‐inflammatory cytokines by inactivating MAPK‐NF‐kB signaling. The normalized function was also accompanied by the overexpression of FXR and AHR, which are implicated in gut microenvironmental homeostasis.

Of particular, the probiotic‐derived regulation of the multiple molecular factors was tightly linked to intestinal metabolomic reprogramming as a major mode of action, which was especially featured by increased SCFAs and indoles. Besides, we linked the metabolomic and metagenomic characteristics of the mouse model to the ones of NAFLD‐ELE patients, which assured the key metabolic signatures in common and led to the discovery of potential biomarker, precisely diagnosing NAFLD progression.

A growing evidence has demonstrated interactive causation of gut microbiota‐derived metabolites as active modulators, including tryptophan metabolites and SCFAs, which encompasses diet, microorganisms and host.^27^, ^33^ Tryptophan metabolites, in particular, have been shown to affect the development of NAFLD accompanied by compositional alteration of gut microbiota.^4^, ^34^ Indole compounds are major products of tryptophan‐derived metabolites, including indole‐3‐acrylic acid, indole‐3‐acetic acid and indole‐3‐propionic acid.^4^, ^6^, ^28^ In a previous study, indole‐3‐acrylic acid was shown to promote intestinal epithelial barrier function and to mitigate inflammatory responses via NRF2 activation.^27^ Similarly, indole‐3‐acetate was reported to attenuate inflammatory response of macrophages and cytokine‐mediated lipogenesis in hepatocytes.^28^ Indole‐3‐propionic acid has been found to improve HFD‐induced intestinal epithelial barrier damage and to inhibit endotoxin‐induced production of pro‐inflammatory cytokines via inactivation of NF‐κB signaling.^35^ Indoles have also been proposed to have agonistic activity for AHR, enhancing gut barrier integrity,^36^ suppressing inflammation^37^ and maintaining metabolic homeostasis.^38^ Accordingly, our results demonstrated the over‐expression of AHR particularly, in the LL group, whose levels were substantially higher than all the other groups. The hyper‐regulation may partly explain the better performance of the LL group on the protective effects compared to the PP group. Indeed, our metabolomic analysis with functional validation assured the pivotal role of the indole compounds for NAFLD. The caecal metabolomics demonstrated that the indole compounds were normalized in the mice fed by LL and PP. Remarkably, indole‐3‐propionic acid showed the consistent and significant changes in two probiotics‐fed groups compared to the WD group (WD). The spatially‐resolved profiles of the hepatic and sera contents evidenced the direct effect of the compound on the NAFLD progression.

BAs have direct or indirect antimicrobial effects thus modulate the composition of the microbiota, which in turn affects the size and composition of BA pool.^39^ FXR exerts tissue‐specific regulation of BA synthesis and transport.^40^ Previous studies showed that mice with FXR deficiency were afflicted with hepatic steatosis as well as glucose and insulin intolerance, the main hallmarks of NAFLD in humans.^41^, ^42^ There have been incoherent results regarding BAs, due to the generic complexity of BA metabolism and non‐identical experimental settings (e.g., different spatial and temporal examinations). Nonetheless, the WD‐induced NAFLD in our study was best characterized by dysregulation of unconjugated BAs, consistent with other recent studies.^43^ Further, our results clearly showed that the WD‐induced dysregulation of BAs was largely restored by the strain‐administration. The faecal BAs in the patients with NAFLD‐ELE did not share identical pattern compared to those of WD‐induced NAFLD mouse model; however, the general trend was identified in the alteration of unconjugated BAs by WD. In particular, the diet‐induced accumulation of taurocholic acid has been reported to perturb gut microbial symbiosis in a mouse model^44^, ^45^ and to stimulate hepatic inflammation and fibrosis.^46^

Despite well‐known beneficial effects, the functional roles of SCFAs remain controversial, particularly in metabolic disorders, including NAFLD. Previous studies have shown the deleterious effects of SCFAs on the NAFLD patients.^47^, ^48^ To the contrary, our result demonstrated confirmative patterns, the significant depletion of all major SCFAs (acetic acid, propionic acid and butyric acid) in mouse model and patients with NAFLD‐ELE. Moreover, the caecal levels were substantially normalized in the probiotics‐fed groups compared to the WD group. The discrepancy (heterogeneity) in the NAFLD patients may be at least partly explained by population variability in geography, metabolic diseases and related medication as reviewed in a few literatures. Although our animal experiment was not designed for time course, sequential mode of action may be expected as follows: WD induces severe reduction of microbial diversity, which leads to the depleted SCFAs content^49^ triggering accelerated glucose consumption by host enterocytes and colonocytes to compensate for depletion of the main energy sources. Accordingly, we observed a decreased glucose level in the WD group, which was ameliorated by strains supplementation, resulting in glucose levels equivalent to those in the normal control group. It is also worth noting that a significantly higher content of mannose was identified in the normal control and the strain‐fed groups. The beneficial effect of dietary supplementation of mannose is associated with the modulation of gut microbiome, leading to prevention of diet‐induced obesity and amended host metabolism.^50^

Note that the key metabolic signatures in the mouse model corresponded to the ones in the NAFLD‐ELE patients, which rationalized the biomarker application. Accordingly, the diagnostic model showed the highest level of discrimination power (AUC, 0.918–0.922). The subsequent functional validation based on in vitro (LPS‐treated macrophage) and in vivo (NAFLD mouse) models demonstrated that three indole compounds were causally linked to the suppression of proinflammatory cytokines such as TNF‐α and IL‐1β. These results corroborated the protective effect of a diet supplemented with *L. lactis* and *P. pentosaceus* against NAFLD progression via the production of the indole metabolites.

It is noteworthy that NAFLD progression was more effectively inhibited by the LL‐intervention compared to PP‐treatment. Although the overall similarity was determined in both strain‐therapies, the clinical parameters, particularly ALT level, serum endotoxin level and adiponectin were more significantly improved by the LL‐treatment. Aforementioned overexpression of AHR by the LL‐supplementation may in part explain the outperformance on gut barrier integrity, which leads to more effective ameliorative action on serum endotoxin level. AHR has also been proposed to have anti‐inflammatory activity, which may affect the normalized level of ALT as determined in our current result. Nonetheless, we did not catch the metabolites that induce the differential expression of AHR directly. SCFAs are known to act as the agonists and indoles as well, whose abundance was overall equivalent between two probiotics‐treatments. Alternatively, we observed the significant differences in metabolites that may explain the differential bioactivity. The metabolites included *N*‐acetlysphingosine, bilirubin, betulin and 4‐phenylbutyric acid. *N*‐acetylsphingosine was shown the highest fold‐difference between two treatment‐groups, known to inhibit the accumulation of lipid droplets by promoting fatty acid oxidation.^51^ Likewise, bilirubin was reported to reduce hepatic fat accumulation by promoting the transcriptional activity of peroxisome proliferator‐activated receptor α (PPARα).^52^ Betulin has been linked to inhibitory activity on fat accumulation and pro‐inflammatory response by increasing the expression of PPARα plus suppressing NF‐κB and SREBP‐1.^53^, ^54^ 4‐phenylbutyric acid has been found to inhibit hepatic lipogenesis by inhibiting the upstream lipogenic transcriptional factors SREBP‐1c and XBP1.^55^

Our current study has some limitations as follows: We did not justify the quantitative changes of the probiotics that were administered to mouse since the genetic components were not detected in caecal samples, which may limit the accurate estimation of the treatment efficacy for future application. The limitation may be overcome by investigating targeted bacterial taxa through deep amplicon sequencing of the phylogenetic marker gene in the different parts of gastrointestinal tract with a subdivided multi‐time point longitudinal study design. Following study should also encompass the comprehensive characterization of the microbial compositional changes, including the identification of keystone microbiome that conferred the overproduction of indole compounds in the current study.

In summary, our current investigation proposed the mechanism underlying the protective effects of the probiotics, *L. lactis* and *P. pentosaceus* on NAFLD progression. The integrative profiling of the caecal metabolome in NAFLD mouse model demonstrated the overall normalization of the gut microbial metabolites by the probiotics‐supplementation (e.g., SCFAs, indole metabolites and BAs). And the subsequent functional studies based on in vitro and in vivo models justified the pivotal and causative roles of the keystone metabolites, indole derivatives. Besides, the cross‐validation against the faecal metabolome of NAFLD patients suggested the potentials of the development of non‐invasive personal biomarkers and novel therapeutics.

## CONFLICT OF INTEREST

Byung Yong Kim was employed by company ChunLab, Inc. Byoung Kook Kim was employed by company Chong Kun Dang Bio. All other authors attest that there are no commercial associations that might be a conflict of interest in relation to the submitted manuscript.

## Supporting information

Supporting Information

## Data Availability

The data that supports the findings of this study are available in the supplementary material of this article.
