# Supplementary material for: Lactobacillus lactis and Pediococcus pentosaceus‐driven reprogramming of gut microbiome and metabolome ameliorates the progression of non‐alcoholic fatty liver disease
Source: Clin Transl Med. 2021 Dec 29;11(12):e634. doi: 10.1002/ctm2.634 (PMC8715831; doi:10.1002/ctm2.634)
Supplement: Supplementary file 1 — Supporting Information [file CTM2-11-e634-s001.docx]

**Supplementary methods**

***Lactobacillus Lactis and Pediococcus Pentosaceus-driven Reprogramming of Gut Microbiome and Metabolome Ameliorates the Progression of Non-alcoholic Fatty Liver Disease***

**Strain preparation**

*Lactobacillus* and *Bifidobacterium* used for the preliminary study were isolated from various sources such as sour milk, cheese, healthy adult stool and new-born’s feces. *L. lactis* is lactic acid bacterium that was isolated from sour milk. *P. pentosaceus* (KCTC 18308P) is a strain of lactic acid bacterium that was extracted from finger millet (*Eleusine coracana*) gruel. *L.* *lactis* and *P. pentosaceus* were grown into a flask containing de Man, Rogosa, and Sharpe media (BD/Difco) and incubated under anaerobic conditions (37°C for 24 h). Stocks of strains were produced by mixing the culture broth with an equivalent 20% skim milk solution and then storing the mixture at -80°C.

The seed culture was grown in a flask containing MRS broth for *L. lactis* and *P. pentosaceus*. Broth was inoculated in an optimized media in a fermenter (Bio Control & Science, MARADO-05D-PS). The fermentation was performed with a constant condition (pH 5.5~6.0 by automatically adding NaOH solution (25% w/v) with 120 rpm agitation at 37°C for 18~20 h). At the final stage of fermentation, the strains were harvested by centrifugation at 6000 rpm for 10 min (Hanil, Supra R12). The lyophilization of concentrated strains (40X) was completed in accordance with the manual (Cooling & Heating System, Lab-Mast 10). After lyophilization, serial dilution method was used for the measure of colony-forming units (CFU)/g of strains powder. Strains were suspended in 0.1 M PBS and adjusted to a density of 10^9^ CFU/mL prior to use.

**STAM mouse model**

Pathogen-free 14-day pregnant C57BL/6J mice were purchased from Dooyeol Biotech (Seoul, Korea). STAM-NASH model was induced in male mice by a single subcutaneous injection of 200 μg STZ (Sigma, MO, USA) at 2 days after birth and feeding with HFD32 ad libitum after 4 weeks of age. Probiotics were suspended in DPBS and oral gavage feeding at a concentration of 10^9^ CFU/g for 3 weeks (2 times/week) after 6 weeks of age.

**Animal sacrifice**

The animals were sacrificed via inhalation anesthesia overdose (isoflurane, Aerane; Baxter, Deerfield, IL, USA) at the end of the treatment period. They were weighed, and the blood, liver, stool, and small intestine were collected. Whole blood (800 μL) samples were centrifuged (19,000 × *g* for 5 min) to collect serum. Liver and stool were rapidly excised and stored at ­80°C.

**Pathology classification**

Specimens were fixed with 10% formalin after extraction and routinely embedded in paraffin, and prepared sections were stained with hematoxylin and eosin, Masson's trichrome, and CD 68. NAFLD activity score (NAS), sum of scores for steatosis, lobular inflammation, and ballooning, was assessed.^1^ All liver specimens were analysed by a hepato-pathologist (S. H. H.).

Steatosis and inflammation were calculated according to the NASH clinical research network scoring system for NAFLD. Steatosis was divided into 4 grades (0: <5%, 1: 5%~33%, 2: 34%~66%, and 3: >66% of steatosis). Inflammation was classified to 4 4 stages (0: none, 1: 1~2 foci/×20, 2: 2~4 foci/×20, and 3: >4 foci/×20 field).

For the macrophage staining, CD 68 stain was completed by the immunohistochemistry. Formalin-fixed and paraffin-embedded liver tissues were stained in the Bond-Max automatic immunostaining device (Leica Biosystem, Newcastle, UK) using a bond polymer intensity detection kit (Leica Biosystem). Cluster of differentiation 68 (CD 68; Leica Biosystem, Newcastle, UK, RTU) were utilized as an antibody. Counterstaining was performed with Harris hematoxylin. CD68 immunohistochemical stains were analysed with an average field of ⅹ200 and ⅹ400 by using automatic imaging program.

**Real-time reverse transcription-polymerase chain reaction analysis**

Procedures were performed according to the manufacturer’s instructions. A trizol reagent kit (Invitrogen, Gaithersburg, MD, USA) was utilized for the isolation of tissue RNA. A cDNA reverse transcription kit (Applied Biosystems, Foster City, CA) was used for the cDNA. Prepared cDNA was amplified for quantitative polymerase chain reaction (PCR) with reagents [the Luna® Universal Probe qPCR Master Mix (New England Biolabs Beverly, MA, USA) and each target specific probe-primer (Applied Biosystems, Foster City, CA)].

**Enzyme-linked immunosorbent assay**

Liver homogenates were incubated with assay solution (PRO-PREP™ Protein Extraction Solution, iNtRON Biotechnology, Korea) at 4 °C for 30 min. Homogenates were vortexed for 3 min with stainless bead and centrifuged at 10,000 g for 10 min. Harvested supernatants were analyzed for pro-inflammatory cytokines such as TNF-α, IL-1β, and IL-6. Enzyme-linked immunosorbent assay (R&D Systems, Minneapolis, MN) was completed according to the manufacturer’s instructions.

**Western blots**

PRO-PREP™ Protein Extraction Solution (iNtRON Biotechnology, Korea) was used for the incubation of tissue homogenates (ice for 30 min). We performed vortexing for 3 min after added stainless bead and centrifuging at 10,000 g for 10 min. Protein (30 ㎍) was resolved by 10% SDS-PAGE and transferred to nitrocellulose membranes. The blots were probed with the indicated primary antibodies RBP4 (1:1000, Abcam, Cambridge, MA, USA), phospho-NF-κB p65, MAPKs, phosphor-MAPKs, and GAPDH (1: 1000, Cell Signaling Technology, Beverly, MA, USA) followed by incubation with the corresponding horseradish peroxidase-conjugated secondary antibodies (1:10000 dilution). The membrane was reacted with the enhanced chemiluminescence (ECL) substrate solution and analyzed by Amersham Imager 680 (GE Healthcare UK Ltd, Buckinghamshire, UK).

**Endotoxin level in feces**

The matched feces samples were used for endotoxin measurement and 16S rRNA gene sequencing. We collected animal feces on a day of euthanasia according to a previously described protocol. Briefly, the feces were suspended in sterile PBS to a concentration of 50 mg per 500 μL and vortexed softly to avoid disruption of strains. After centrifuging fluids for 15 min at 3000 rpm, the collected supernatant was filtered with 0.45-μm and 0.22-μm filter. Filtered fluids were inactivated for 15 min at 90°C and stored at ‒80°C.

The feces endotoxin were analysed by using a limulus amoebocyte lysate assay (No. 50-647U; Lonza Inc) according to the manual.^2^ The feces supernatant was mix with sterile water (10,000-fold dilution) and incubated for 15 min at 90°C.

**Trans-epithelial electrical resistance assay**

For the trans-epithelial electrical resistance (TEER) measurements, Caco-2 cells were used as described previously.^3^ Cells were inoculated into transwell-clear inserts (12-well clusters, 6.5-mm inserts with polyester membrane, pore diameter 0.4 μm, Corning NY) at a density of 10^5^ cells/insert. Each insertion was located on top of a well in plates (1 ml-bottom and 200 μl media-top). Caco-2 cells were incubated for 5 days until confluence in Minimum Essential Medium Eagle with 20% fetal bovine serum without antibiotic-antimycotic (Gibco, Carlsdad, CA, USA) at 37°C in a humidified 5% atmosphere.

TEER measurements were performed using a Millicell Electrical resistance system (Millipore, Billerica, MA, USA). When monolayer of cells reached the confluence, Caco-2 cells were co-incubated with 200 μl of OD_600_ 0.3 bacterial suspension (7 ⅹ 10^7^ CFU/ml) in MEM media for 8 hrs and the TEER was measured respectively. TEER values were compared with those of the control and expressed as the ratio of the TEER value of sample after 8 hr co-incubation to the TEER value of control after 8 hr co-incubation.

**Reference**

1. Kleiner DE, Brunt EM, Van Natta M, et al. Design and validation of a histological scoring system for nonalcoholic fatty liver disease. *Hepatology*. Jun 2005;41(6):1313-1321.

2. Yoshida N, Emoto T, Yamashita T, et al. Bacteroides vulgatus and Bacteroides dorei Reduce Gut Microbial Lipopolysaccharide Production and Inhibit Atherosclerosis. *Circulation*. Nov 27 2018;138(22):2486-2498.

3. Anderson RC, Cookson AL, McNabb WC, et al. Lactobacillus plantarum MB452 enhances the function of the intestinal barrier by increasing the expression levels of genes involved in tight junction formation. Research Support, Non-U.S. Gov't. *BMC Microbiol*. Dec 9 2010;10:316.

**Supplymentary Figures**

**
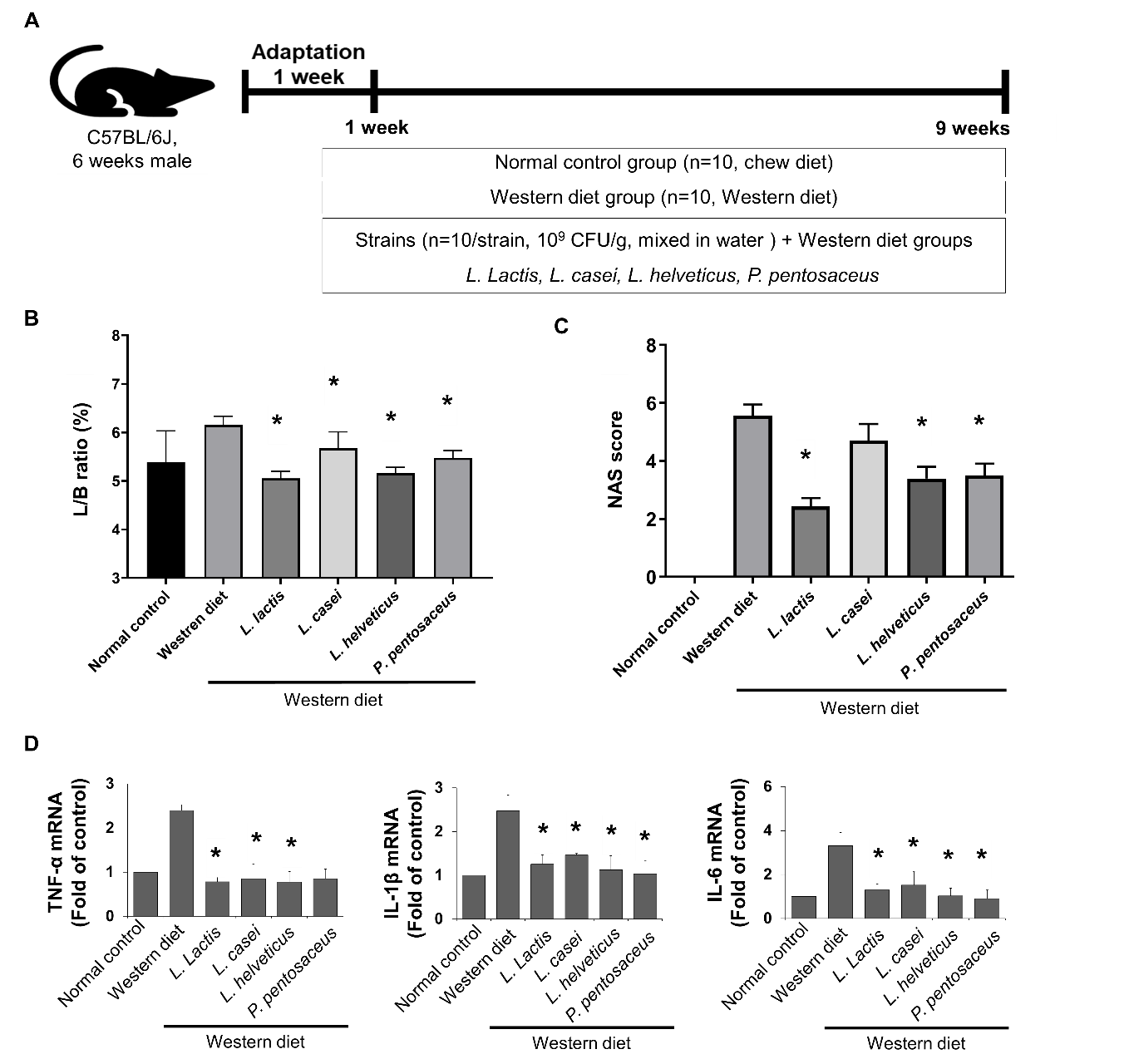
**

**Supplementary Figure1. Effects of probiotics in Wstern diet-induced NAFLD model.** (A) Flow chart of the animal experiment. (B) Effect of dietary on liver/body weight ratio in mice. Compared with mice fed Western diet. (C) NAFLD Activity Score (NAS). (D) The levels of inflammatory cytokines mRNA (TNF-α, IL-1β, and IL-6) in the mouse liver. All data are shown as mean ± SEM of experiments. one-way ANOVA, the Kruskal-Wallis and Friedman tests was used for all statistical analyzes. *p<0.05 as compared with Western diet.

**
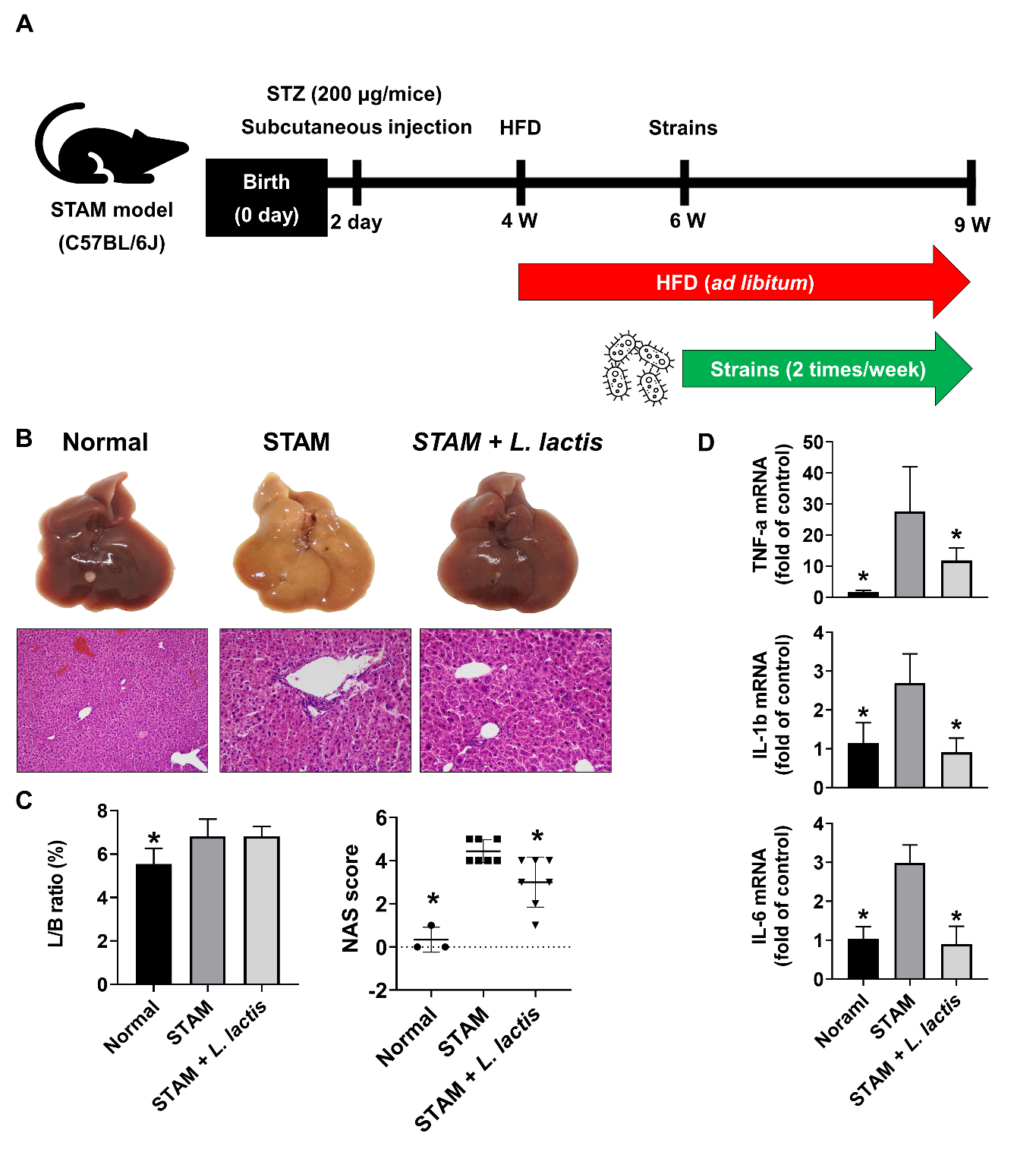
**

**Supplementary Figure 2. Effects of *L. lactis* in STAM-non-alcoholic fatty liver disease model.** (A) Flow chart of the animal experiment. (B) Gross specimen of mice liver. Representative photographs of liver samples were shown. (C) Effect of dietary on liver/body weight ratio and NAS in mice. (D) The levels of inflammatory cytokines mRNA (TNF-α, IL-1β, and IL-6) in the mouse liver. All data are shown as mean ± SEM of experiments. one-way ANOVA, the Kruskal-Wallis and Friedman tests was used for all statistical analyzes. *p<0.05 as compared with STAM-NASH group.

**
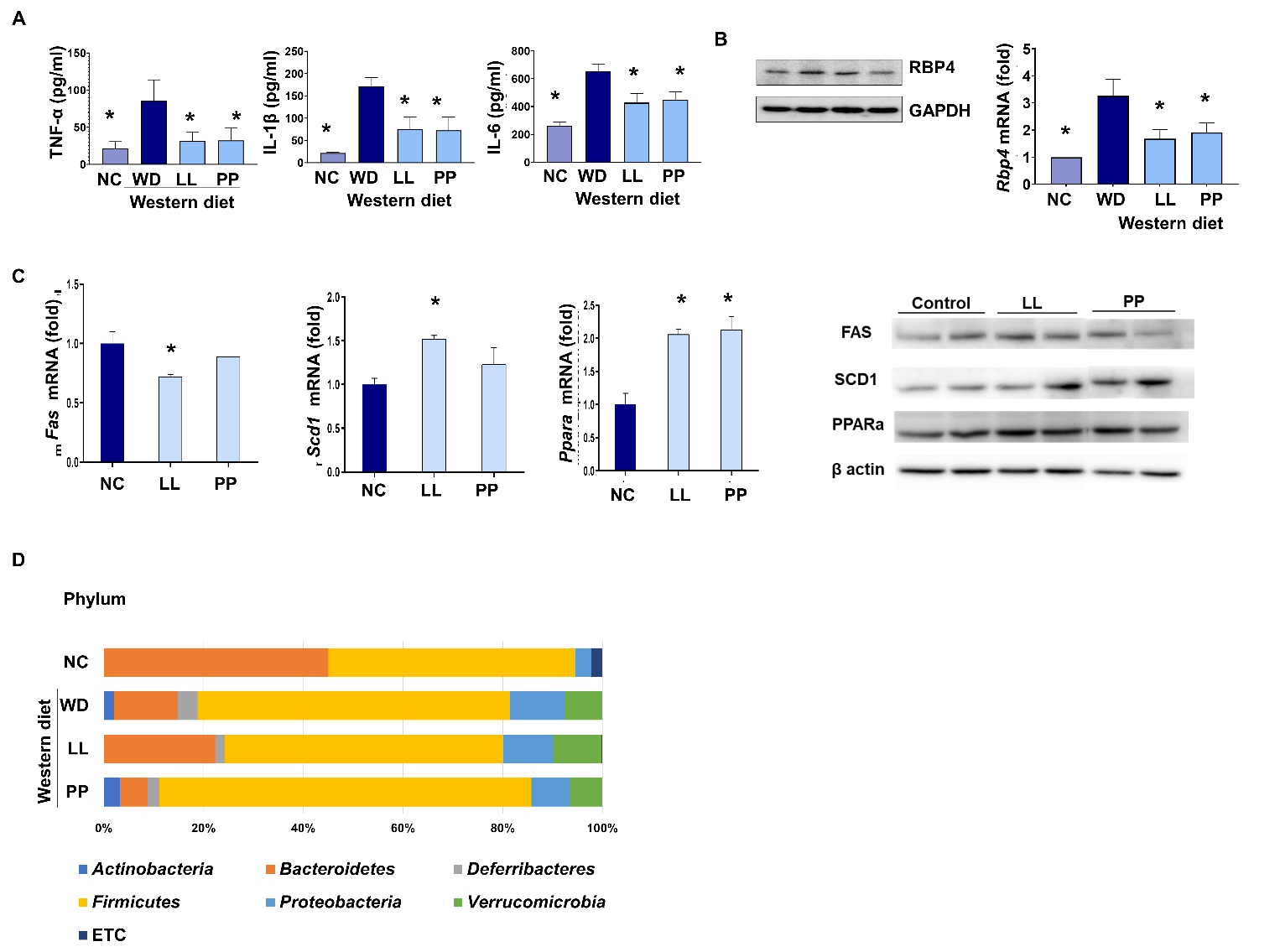
**

**Supplementary Figure 3. *L. lactis* and *P. pentosaceus* suppress the progression of non-alcoholic fatty liver disease by modulating gut-liver axis.** (A) The levels of inflammatory cytokine TNF-α, IL-1β, and IL-6 in the mouse liver (n=5). Inflammatory cytokine protein using ELISA. (B) The level of retinol binding protein 4 (RBP4) protein and mRNA analysis using Western blotting and qRT-PCR. Mouse liver analyzed for the RBP4 and GAPDH by Western blotting using specific antibodies. (C) Lipid metabolism associated genes. (D) Differential microbial composition at phylum analysis.

**
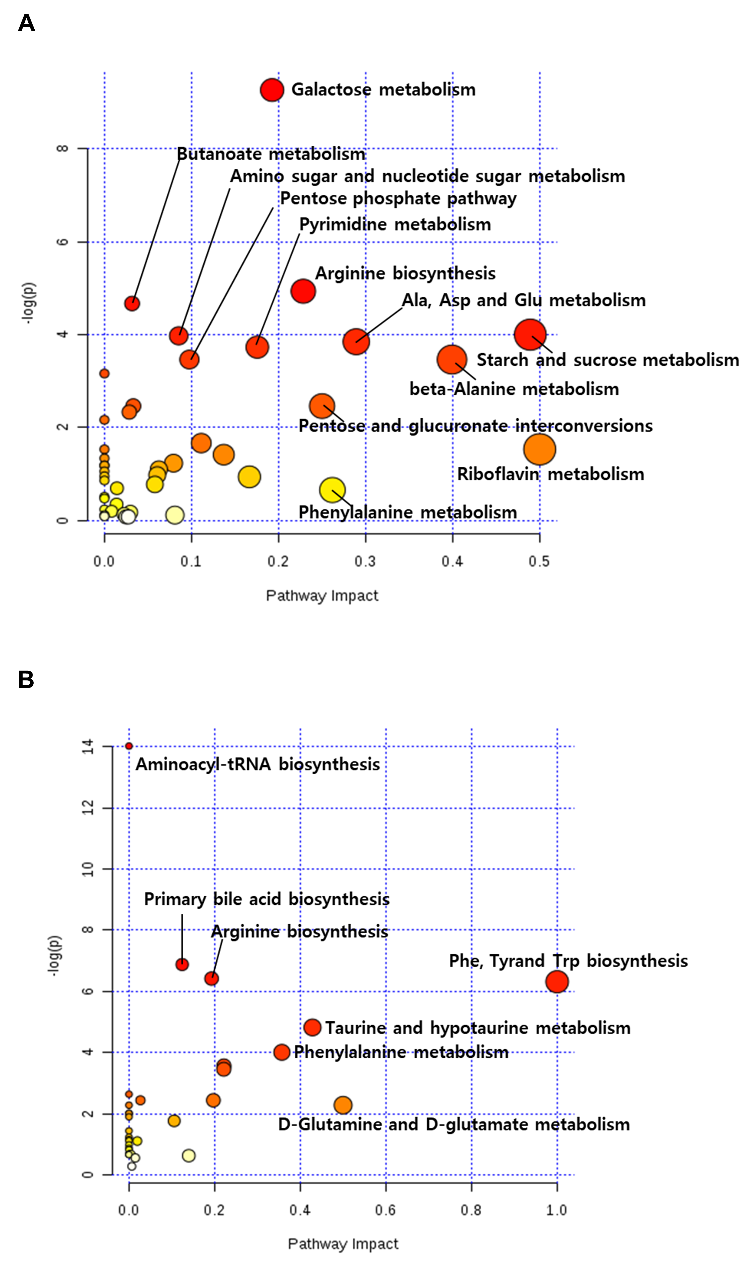
**

**Supplementary Figure 4. Pathway over-representation anlysis of cecal metabolites.** Pathway over-representation analysis was performed on list of metabolites name. X-axis means pathway impact which was calcuated in accordance with the betweenness of a node. Y-axis indicates the significance level. Node size and color are expressed in proportion to the pathway impact and statistical significance level, respectively. The list included the metabolites with statistical significance by student t test (p < 0.05). (A) Present up-regulation metabolites in NC against WD (B) Down-regulation in NC against WD.

**
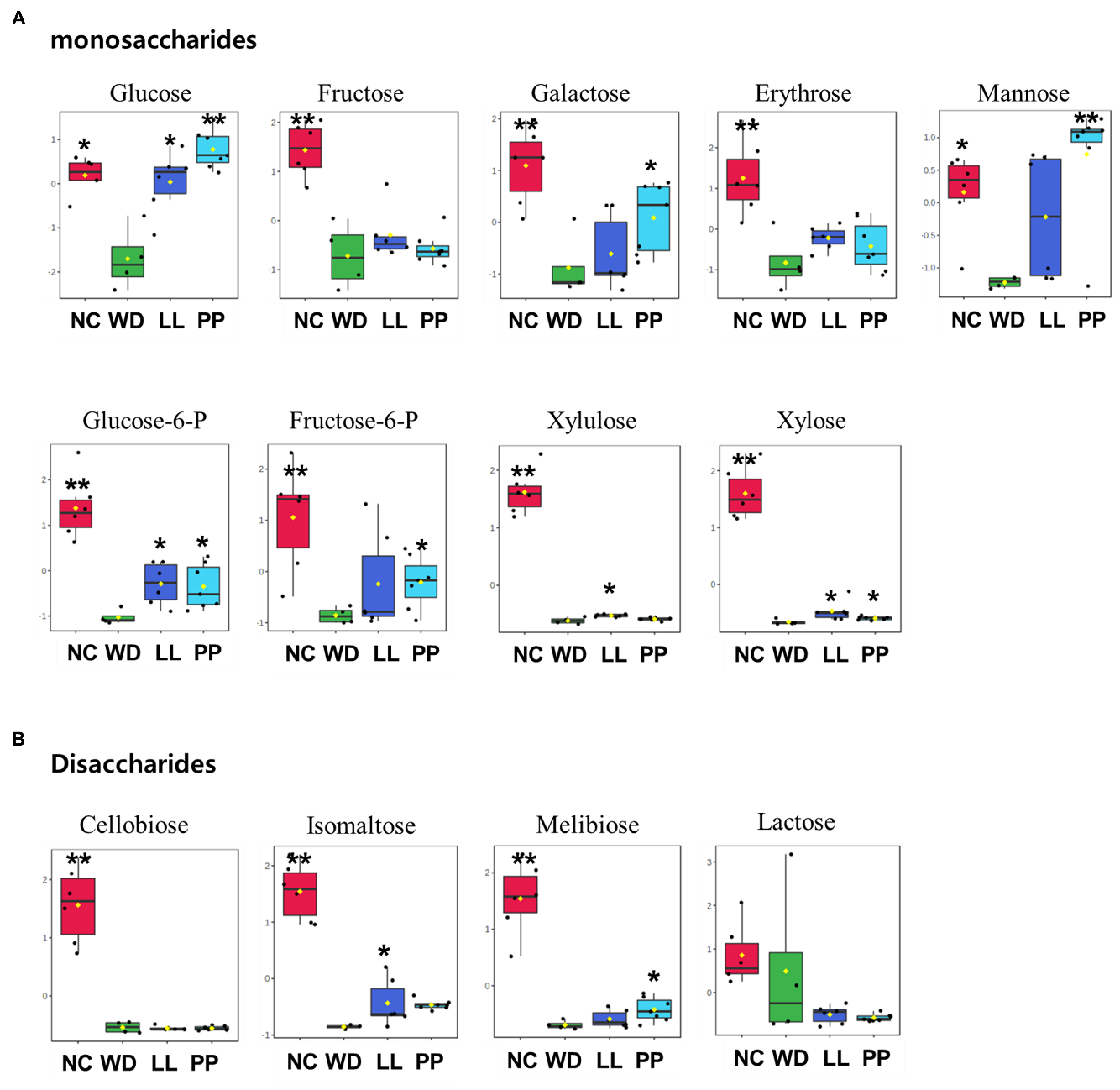
**

**Supplementary Figure 5. Box plot of cecal carbohydrates.** Box plot of 9 monosaccharides (A) and 4 disaccharides (B). Y-axis indicates relative abundance. * indicates the statistical significance against WD group by Mann-Whitney U test (p <0.05). ** indicates the statistical significance against WD group by nonparametric Kruskal-Wallis test and Dunn’s test adjusted by Benjamini–Hochberg correction (p <0.05).

**
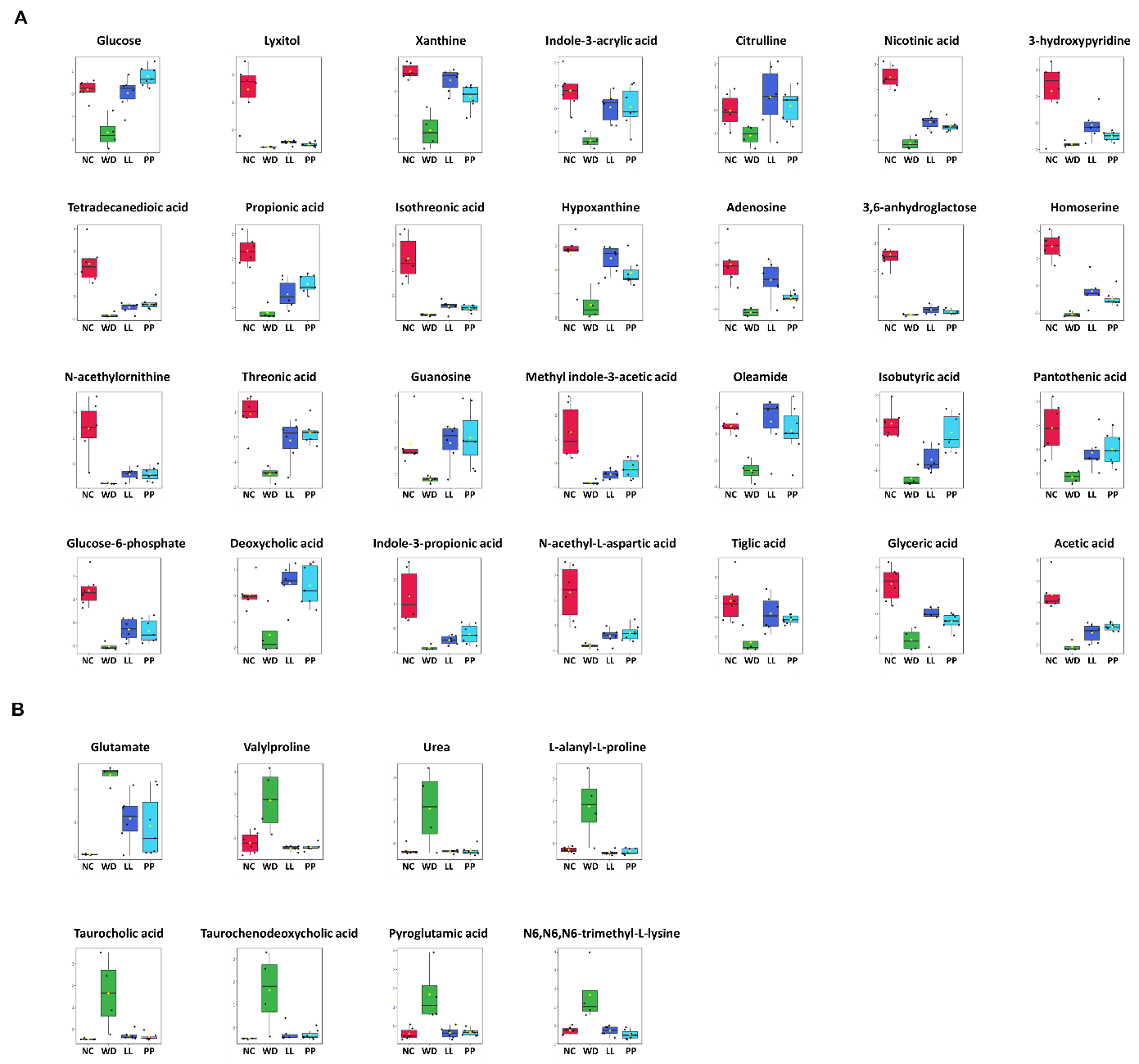
**

**Supplementary Figure 6. Common metabolites of cecal sample according to pairwise t test.** Box plot of cecal metabolites which have common abundance pattern in NC, LL and PP groups against WD. Y-axis indicates relative abundance. (A) Significantly up-regulation in NC, LL and PP groups compare to WD. (B) Significantly down-regulation in NC,LL and PP groups compare to WD.

**
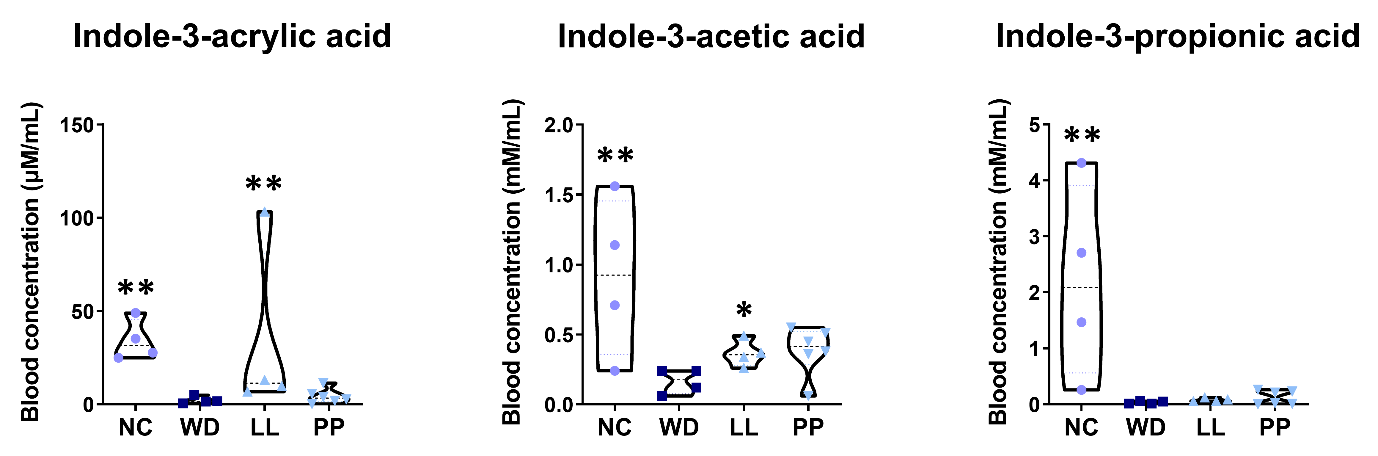
**

**Supplementary Figure 7. Concentrations of indole compounds in mouse serum.** * indicates the statistical significance against WD group by Mann-Whitney U test (p <0.05). ** indicates the statistical significance against WD group by nonparametric Kruskal-Wallis test and Dunn’s test adjusted by Benjamini–Hochberg correction (p <0.05).

**Supplementary Table 1. Summary of preliminary animal study**

| Biomarker | Group (n=10/each) | Mean ± SD | p value* |
| --- | --- | --- | --- |
| Liver/body weight ratio | WD (8 weeks) | 6.2 ± 0.6 | - |
|  | WD + *L. lactis* | 5.1 ± 0.5 | <0.001 |
|  | WD + *L. helveticus* | 5.2 ± 0.4 | <0.001 |
|  | WD + *L. acidophilus* | 5.5 ± 0.4 | <0.001 |
|  | WD + *P. pediococcus* | 5.5 ± 0.5 | 0.009 |
|  | WD + *L. paracasei* | 5.7 ± 0.4 | 0.032 |
| Histology score  (fatty change / inflammation) | WD (8 weeks) | 3.1 ± 0.3 / 2.0 ± 0.0 | - |
|  | WD + *L. lactis* | 0.7 ± 0.7 / 0.7 ± 0.7 | <0.001 |
|  | WD + *P. pediococcus* | 1.3 ± 0.5 / 1.4 ± 0.5 | <0.001 |
|  | WD + *L. helveticus* | 1.3 ± 0.5 / 1.7 ± 0.7 | <0.001 |
|  | WD + *L. acidophilus* | 1.6 ± 0.7 / 1.8 ± 0.6 | <0.001 |
|  | WD + *L. casei* | 1.8 ± 0.4 / 2.1 ± 0.6 | <0.001 |
|  | WD + *L. plantarum* | 1.8 ± 0.6 / 2.3 ± 0.7 | 0.003 |
|  | WD + *L. paracasei* | 1.6 ± 0.5 / 2.1 ± 0.9 | 0.035 |
|  | WD + *B.bifidum* | 1.6 ± 0.5 / 2.5 ± 0.8 | 0.037 |
|  | WD + *L. fermentum* | 1.8 ± 0.4 / 2.7 ± 0.5 | 0.042 |

N, number; SD, standard deviation; WD, Western diet

*compared with WD group

**Supplementary Table 2. The index pairs and sequences used for 16S rRNA gene amplicon sequencing of each sample**

| Sample | i5 index name | i5 sequence | i7 index name | i7 sequence |
| --- | --- | --- | --- | --- |
| H110 | S517 | GCGTAAGA | N710 | CGAGGCTG |
| H209 | S513 | TCGACTAG | N720 | CGGAGCCT |
| H255 | S517 | GCGTAAGA | N715 | ATCTCAGG |
| H314 | S515 | TTCTAGCT | N722 | ATGCGCAG |
| H323 | S517 | GCGTAAGA | N719 | GCGTAGTA |
| H428 | S517 | GCGTAAGA | N722 | ATGCGCAG |
| H59 | S511 | TCTCTCCG | N727 | CGATCAGT |
| H64 | S511 | TCTCTCCG | N728 | TGCAGCTA |
| chun-01 | S517 | GCGTAAGA | N701 | TAAGGCGA |
| chun-11 | S517 | GCGTAAGA | N710 | CGAGGCTG |
| chun-45 | S506 | ACTGCATA | N701 | TAAGGCGA |
| me109 | S511 | TCTCTCCG | N705 | GGACTCCT |
| me122 | S511 | TCTCTCCG | N706 | TAGGCATG |
| me655 | S511 | TCTCTCCG | N726 | CCTAAGAC |
| wards186 | S513 | TCGACTAG | N722 | ATGCGCAG |
| wards193 | S513 | TCGACTAG | N724 | ACTGAGCG |
| wards243 | S502 | CTCTCTAT | N729 | TCGACGTC |
| wards29 | S513 | TCGACTAG | N705 | GGACTCCT |
| wards38 | S513 | TCGACTAG | N706 | TAGGCATG |
| wards44 | S513 | TCGACTAG | N710 | CGAGGCTG |
| wards5 | S511 | TCTCTCCG | N728 | TGCAGCTA |
| wards65 | S513 | TCGACTAG | N718 | GGAGCTAC |
| wards67 | S513 | TCGACTAG | N719 | GCGTAGTA |
| wards7 | S511 | TCTCTCCG | N729 | TCGACGTC |
| wards9 | S513 | TCGACTAG | N701 | TAAGGCGA |
| 4th-1245 | S505 | GTAAGGAG | N702 | CGTACTAG |
| 4th-19-1 | S518 | CTATTAAG | N710 | CGAGGCTG |
| 4th-19-2 | S520 | AAGGCTAT | N710 | CGAGGCTG |
| 4th-2 | S507 | AAGGAGTA | N702 | CGTACTAG |
| 4th-239 | S511 | TCTCTCCG | N703 | AGGCAGAA |
| 4th-367 | S520 | AAGGCTAT | N704 | TCCTGAGC |
| 4th-369 | S521 | GAGCCTTA | N704 | TCCTGAGC |
| 4th-373 | S522 | TTATGCGA | N704 | TCCTGAGC |
| 4th-393 | S507 | AAGGAGTA | N705 | GGACTCCT |
| 4th-397 | S508 | CTAAGCCT | N705 | GGACTCCT |
| 4th-409 | S521 | GAGCCTTA | N705 | GGACTCCT |
| 4th-429 | S507 | AAGGAGTA | N701 | TAAGGCGA |
| 4th-463 | S508 | CTAAGCCT | N701 | TAAGGCGA |
| 4th-499 | S510 | CGTCTAAT | N701 | TAAGGCGA |
| 4th-543 | S513 | TCGACTAG | N701 | TAAGGCGA |
| 4th-569 | S518 | CTATTAAG | N701 | TAAGGCGA |
| 4th-572 | S520 | AAGGCTAT | N701 | TAAGGCGA |
| 4th-637 | S521 | GAGCCTTA | N701 | TAAGGCGA |
| 4th-646 | S522 | TTATGCGA | N701 | TAAGGCGA |
| 4th-703 | S503 | TATCCTCT | N702 | CGTACTAG |

**Supplementary Table 3. The comparative abundance of microbial genes related to selected metabolic pathways and signaling pathways of mouse stool**

| Definition | NC | WD | LL | PP |
| --- | --- | --- | --- | --- |
| Biosynthesis of secondary metabolites | 4.755 | 5.439 | 5.349 | 5.881 |
| Biosynthesis of antibiotics | 3.521 | 4.009 | 3.953 | 4.373 |
| Two-component system | 2.101 | 2.831 | 2.691 | 2.485 |
| Carbon metabolism | 1.617 | 1.810 | 1.775 | 1.989 |
| Biosynthesis of amino acids | 1.604 | 2.360 | 2.224 | 2.649 |
| Purine metabolism | 1.457 | 1.593 | 1.581 | 1.791 |
| Ribosome | 1.417 | 1.511 | 1.474 | 1.701 |
| Quorum sensing | 1.162 | 1.486 | 1.396 | 1.530 |
| Pyrimidine metabolism | 1.068 | 1.191 | 1.177 | 1.379 |
| Amino sugar and nucleotide sugar metabolism | 0.993 | 1.134 | 1.141 | 1.352 |
| Pyruvate metabolism | 0.802 | 0.840 | 0.824 | 0.908 |
| Porphyrin and chlorophyll metabolism | 0.706 | 0.602 | 0.613 | 0.501 |
| Sphingolipid metabolism | 0.661 | 0.404 | 0.479 | 0.329 |
| Starch and sucrose metabolism | 0.654 | 0.820 | 0.817 | 1.145 |
| Glycerophospholipid metabolism | 0.602 | 0.533 | 0.528 | 0.552 |
| Glycolysis / Gluconeogenesis | 0.592 | 0.787 | 0.783 | 1.016 |
| Base excision repair | 0.576 | 0.429 | 0.432 | 0.449 |
| Degradation of aromatic compounds | 0.564 | 0.369 | 0.402 | 0.344 |
| Aminoacyl-tRNA biosynthesis | 0.545 | 0.635 | 0.612 | 0.759 |
| Cysteine and methionine metabolism | 0.545 | 0.643 | 0.628 | 0.754 |
| Tryptophan metabolism | 0.536 | 0.359 | 0.383 | 0.240 |
| Arginine and proline metabolism | 0.531 | 0.477 | 0.487 | 0.433 |
| Benzoate degradation | 0.525 | 0.331 | 0.373 | 0.259 |
| Alanine, aspartate and glutamate metabolism | 0.524 | 0.641 | 0.630 | 0.677 |
| Methane metabolism | 0.524 | 0.633 | 0.607 | 0.651 |
| Valine, leucine and isoleucine degradation | 0.497 | 0.394 | 0.398 | 0.300 |
| Galactose metabolism | 0.488 | 0.614 | 0.643 | 0.786 |
| DNA replication | 0.457 | 0.499 | 0.482 | 0.551 |
| Nicotinate and nicotinamide metabolism | 0.455 | 0.393 | 0.402 | 0.407 |
| Bacterial secretion system | 0.447 | 0.681 | 0.619 | 0.589 |
| Hepatitis C | 0.443 | 0.293 | 0.309 | 0.181 |
| Glyoxylate and dicarboxylate metabolism | 0.440 | 0.564 | 0.547 | 0.541 |
| Homologous recombination | 0.430 | 0.524 | 0.509 | 0.641 |
| Glycerolipid metabolism | 0.425 | 0.357 | 0.372 | 0.441 |
| Pentose and glucuronate interconversions | 0.419 | 0.320 | 0.348 | 0.345 |
| Glycine, serine and threonine metabolism | 0.412 | 0.493 | 0.485 | 0.570 |
| Pentose phosphate pathway | 0.409 | 0.480 | 0.479 | 0.605 |
| Nucleotide excision repair | 0.406 | 0.379 | 0.374 | 0.395 |
| Pathways in cancer | 0.404 | 0.298 | 0.307 | 0.217 |
| Mismatch repair | 0.399 | 0.505 | 0.479 | 0.586 |
| Sphingolipid signaling pathway | 0.388 | 0.177 | 0.203 | 0.123 |
| Human papillomavirus infection | 0.386 | 0.259 | 0.266 | 0.178 |

Data are determine by PICRUst and Kruskal Wallis H test in mouse cecal analysis (p<0.001).

Statistically significant top 42 markers not shown Figure 3E

NC, normal control; WD, Western diet; LL, *L. lactis*; PP, *P. pentosaceus*

**Supplementary Table 4. Summary of chemical classification of mouse cecal metabolite**

| **Super class** | **Class** | **No. of metabolite** |
| --- | --- | --- |
| Benzenoids | Benzene and substituted derivatives | 20 |
| Homogeneous non-metal compounds | Non-metal oxoanionic compounds  Homogeneous other non-metal compounds | 1  1 |
| Lipids and lipid-like molecules | Fatty Acyls  Glycerolipids  Prenol lipids  Steroids and steroid derivatives | 32  1  7  14 |
| Nucleosides, nucleotides, and analogues | 5'-deoxyribonucleosides  Purine nucleosides  Pyrimidine nucleosides  Nucleoside and nucleotide analogues | 1  8  5  1 |
| Organic acids and derivatives | Carboximidic acids and derivatives  Carboxylic acids and derivatives  Hydroxy acids and derivatives  Keto acids and derivatives  Organic carbonic acids and derivatives  Organic phosphoric acids and derivatives  Organic sulfonic acids and derivatives  Organic sulfuric acids and derivatives | 1  60  5  1  1  1  1  1 |
| Organic nitrogen compounds | Organonitrogen compounds | 6 |
| Organic oxygen compounds | Organooxygen compounds | 38 |
| Organoheterocyclic compounds | Caprolactams  Diazines  Imidazopyrimidines  Indoles and derivatives  Lactones  Pteridines and derivatives  Pyridines and derivatives  Quinolines and derivatives  Tetrahydrofurans  Azoles  Pyrrolidines  Imidazopyrimidines  Indolyl carboxylic acids and derivatives  Isoquinolines and derivatives  Tetrapyrroles and derivatives | 1  5  4  6  1  1  5  2  1  1  1  3  2  1  1 |
| Phenylpropanoids and polyketides | Linear 1,3-diarylpropanoids  Phenylpropanoic acids  Cinnamic acids and derivatives  Flavonoids  Isoflavonoids  Phenylpropanoids and polyketides | 3  1  2  5  3  1 |

**Supplementary Table 5.** Summary of univariate statistics on normal control vs Western diet groups

| **Mouse cecal metabolite (up-regulated in WD)** | **p-value** | **FDR** | **Fold change** |
| --- | --- | --- | --- |
| Valine  Palmitic acid  Phenylalanine  Proline  Pipecolic acid  6-chlorohexanol  Uric acid  Lactic acid  Tyrosine  (2R)-2,3-Dihydroxypropanoic acid  Stearic acid  Cytosine  Zymosterol  Creatinine  6-alpha-Prostaglandin I1  Oxoproline  Valylproline  N6,N6,N6-Trimethyl-L-lysine  Asparagine  Histidine  Alanylproline  Taurine  Beta-Leucine  Pyroglutamic acid  Arginine  Urea  Glycocholic acid  2'-Deoxycytidine  Cholesterol  Glutamic acid  Taurochenodeoxycholic acid  Taurocholic acid | 0.026  0.025  0.032  0.001  0.023  0.002  0.020  0.002  0.026  0.016  0.001  0.009  0.004  0.011  0.022  0.007  0.014  0.016  0.018  0.035  0.012  0.004  < 0.001  0.011  0.012  0.027  < 0.001  0.020  0.001  < 0.001  0.012  0.013 | 0.064  0.062  0.073  0.005  0.059  0.015  0.052  0.012  0.064  0.044  0.009  0.031  0.019  0.033  0.057  0.024  0.039  0.044  0.048  0.080  0.034  0.019  0.005  0.034  0.035  0.064  < 0.001  0.051  0.006  < 0.001  0.034  0.036 | 1.197  1.350  1.366  1.579  1.686  1.800  1.802  1.807  1.951  2.072  2.539  2.594  2.689  2.703  2.809  2.905  2.974  3.166  3.747  3.940  4.034  4.625  5.270  6.232  7.720  9.349  15.556  16.616  17.967  33.095  46.447  50.502 |

| **Mouse cecal metabolite (down-regulated in WD)** | **p-value** | **FDR** | **Fold change** |
| --- | --- | --- | --- |
| Dehydroascorbic acid  Isoleucine  Glucose  Citrulline  Sucrose  Squalene  Glycerol-alpha-phosphate  3-phosphoglyceric acid  Fructose  Gamma-aminobutyric acid  Glycerophospho-N-palmitoyl ethanolamine  Uracil  Leucylproline  Erythrose  Oleamide  Pantothenic acid  Galactose  Fucose  Riboflavin  Thymine  Malic acid  Butyrolactam  Fumaric acid  Hexitol  2-Hydroxycaproic acid  Threonic acid  Deoxycholic acid  Xanthine  2,3-dihydroxypyridine  Inositol-4-monophosphate  Guanosine  2,5-dihydroxypyrazine  Glyceric acid  Gluconic acid  Uridine  7-Methyladenine  N-Acetyl-alpha-D-glucosamine  Hypoxanthine  Adenosine-5-monophosphate  Propane-1,3-diol  Sorbitol  Putrescine  Indole-3-acrylic acid  Imidazoleacetic acid  Tiglic acid  2-hydroxypyridine  Spermidine  Fructose-6-phosphate  8-Hydroxyquinoline  Adenosine  Erythritol  Beta alanine  Suberic acid  2-hydroxyhexanoic acid  Nicotinic acid  Shikimic acid  Kynurenic acid  Beta-hydroxybutyric acid  Mannose  Sitosterol  Xylulose  Xylitol  3-hydroxypyridine  Glucose-6-phosphate  N-acetyl-L-aspartic acid  Glutamine  2-deoxytetronic acid  Orotic acid  2-(4-hyrdroxyphenyl)ethanol  Isothreonic acid  19(R)-HETE  Phenylpyruvic acid  Indole-3-acetic acid  Tetradecanedioic acid  N8-Acetylspermidine  Delta-Gluconic acid delta-lactone  N-acetylornithine  Lyxitol  Maltotriose  3-Hydroxypicolinic acid  3-hydroxypropionic acid  3,6-anhydro-d-galactose  Cellobiose  Melibiose  Methyl indole-3-acetic acid  Lactitol  Homoserine  4-Pyridoxic acid  Indole-3-propionic acid  Succinic acid  Azelaic acid  Isomaltose  Xylose  Ethanol phosphate  Lyxose  Enolpyruvic acid  Pelargonidin  Prostaglandin A1 ethyl ester  Ferulic acid  Thiamine  Dodecanedioic acid  Isoferulic acid  5-Hydroxyindole-3-acetic acid | 0.042  0.002  0.001  0.031  0.037  0.011  0.009  0.010  < 0.001  0.002  0.004  0.009  0.001  0.005  < 0.001  0.006  0.002  0.016  0.006  0.009  0.018  0.007  0.004  0.004  0.046  < 0.001  0.008  < 0.001  0.002  0.006  0.019  0.008  < 0.001  < 0.001  0.044  0.035  0.015  0.006  0.003  0.006  < 0.001  0.010  < 0.001  0.028  0.009  < 0.001  0.004  0.007  0.004  0.002  0.033  0.002  0.001  0.012  < 0.001  0.003  0.003  0.01  0.003  < 0.001  < 0.001  < 0.001  0.012  < 0.001  0.005  0.044  0.043  0.003  0.006  0.001  0.029  0.011  0.002  0.001  0.005  0.027  0.004  0.003  0.001  0.001  0.002  < 0.001  < 0.001  < 0.001  0.007  < 0.001  < 0.001  0.003  0.005  0.001  < 0.001  < 0.001  < 0.001  0.049  < 0.001  0.026  0.036  0.015  < 0.001  0.048  < 0.001  0.004  0.004 | 0.093  0.011  0.006  0.071  0.082  0.033  0.031  0.032  0.005  0.013  0.019  0.030  0.011  0.020  0.001  0.023  0.014  0.044  0.024  0.030  0.048  0.024  0.019  0.019  0.099  0.005  0.030  0.002  0.014  0.023  0.050  0.030  0.005  0.002  0.095  0.079  0.04  0.023  0.015  0.023  < 0.001  0.032  0.008  0.067  0.030  < 0.001  0.019  0.024  0.019  0.011  0.077  0.012  0.005  0.035  < 0.001  0.017  0.018  0.033  0.015  0.005  < 0.001  0.002  0.034  0.002  0.022  0.095  0.094  0.016  0.023  0.009  0.068  0.033  0.013  0.008  0.019  0.064  0.019  0.015  0.010  0.011  0.011  0.001  0.003  0.002  0.024  0.001  < 0.001  0.015  0.020  0.006  0.003  < 0.001  < 0.001  0.104  < 0.001  0.064  0.081  0.040  0.001  0.102  0.005  0.019  0.019 | 0.686  0.539  0.519  0.511  0.508  0.505  0.491  0.459  0.434  0.429  0.417  0.406  0.402  0.371  0.365  0.336  0.331  0.329  0.321  0.321  0.314  0.296  0.286  0.285  0.271  0.267  0.265  0.265  0.264  0.256  0.248  0.230  0.225  0.217  0.215  0.209  0.207  0.197  0.196  0.196  0.188  0.182  0.176  0.166  0.155  0.153  0.148  0.144  0.143  0.138  0.137  0.126  0.124  0.12  0.116  0.113  0.109  0.108  0.101  0.100  0.099  0.098  0.094  0.094  0.089  0.088  0.081  0.080  0.068  0.067  0.065  0.061  0.061  0.06  0.057  0.056  0.055  0.053  0.052  0.051  0.05  0.046  0.046  0.044  0.038  0.037  0.037  0.036  0.032  0.028  0.028  0.026  0.016  0.015  0.012  0.010  0.007  0.007  0.006  0.005  0.004  0.003  0.002 |

P-value and FDR were calculated based on Student’s *t*-test

**Supplementary Table 6.** Summary of statistics of carbohydrates.

| **cecum sample** | **Kruskal Wallis test** | | |
| --- | --- | --- | --- |
| **Indole metabolites** | **chi squared** | **p-value** | **FDR** |
| Glucose | 13.313 | 0.004 | 0.009** |
| Xylose | 17.076 | 0.001 | 0.003** |
| Fructose | 13.456 | 0.004 | 0.009** |
| Galactose | 12.949 | 0.005 | 0.009** |
| Mannose | 10.69 | 0.014 | 0.018* |
| Erythrose | 12.216 | 0.007 | 0.010* |
| Glucose-6-phosphate | 16.869 | 0.001 | 0.003** |
| Fructose-6-phosphate | 10.673 | 0.014 | 0.018* |
| Xylulose | 17.629 | 0.001 | 0.003** |
| Cellobiose | 12.811 | 0.005 | 0.009** |
| Isomaltose | 17.283 | 0.001 | 0.003** |
| Melibiose | 15.601 | 0.001 | 0.005** |
| Lactose | 10.505 | 0.015 | 0.018* |
| Maltotriose | 15.042 | 0.002 | 0.005** |

P-value was calculated by Kruskal-Wallis test

*FDR q value<0.05

**FDR q value<0.01

| **Post-hoc** |  | | | | | |
| --- | --- | --- | --- | --- | --- | --- |
| **Indole metabolites** | **NC/WD** | **LL/WD** | **PP/WD** | **NC/LL** | **NC/PP** | **LL/PP** |
| Glucose | 0.104 | 0.089 | 0.002** | 0.798 | 0.124 | 0.117 |
| Xylose | 0.001** | 0.076 | 0.291 | 0.075 | 0.005** | 0.275 |
| Fructose | 0.012* | 0.470 | 0.993 | 0.048* | 0.005** | 0.474 |
| Galactose | 0.012* | 0.581 | 0.093 | 0.013* | 0.210 | 0.163 |
| Mannose | 0.180 | 0.171 | 0.008** | 0.798 | 0.151 | 0.170 |
| Erythrose | 0.012* | 0.512 | 0.589 | 0.034* | 0.015* | 0.743 |
| Glucose-6-phosphate | < 0.001** | 0.093 | 0.090 | 0.034* | 0.026* | 0.875 |
| Fructose-6-phosphate | 0.011* | 0.324 | 0.139 | 0.072 | 0.168 | 0.540 |
| Xylulose | 0.001** | 0.100 | 0.681 | 0.106 | 0.002** | 0.105 |
| Cellobiose | 0.017* | 1.000 | 0.920 | 0.015* | 0.009** | 1.000 |
| Isomaltose | < 0.001** | 0.165 | 0.051 | 0.015* | 0.049* | 0.508 |
| Melibiose | 0.002** | 0.469 | 0.149 | 0.005** | 0.049* | 0.372 |
| Lactose | 0.135 | 0.713 | 0.674 | 0.025* | 0.018* | 0.815 |
| Maltotriose | 0.002** | 0.381 | 0.086 | 0.009** | 0.098 | 0.314 |

Post-hoc was performed by Dunn’s test with Benjamini–Hochberg correction

*adjusted p value<0.05

**adjusted p value<0.01

**Supplementary Table 7. Summary of statistics of short chain fatty acids**

| **Mouse cecal sample** |  |  | | **Kruskal Wallis test** | | |
| --- | --- | --- | --- | --- | --- | --- |
|  | | | **chi squared** | | **p-value** | **FDR** |
| Acetic acid | | | 17.398 | | < 0.001 | 0.001** |
| Propionic acid | | | 17.825 | | < 0.001 | 0.001** |
| Butyric acid | | | 18.177 | | < 0.001 | 0.001** |
| Iso-butyric acid | | | 15.856 | | 0.001 | 0.002** |
| Valeric acid | | | 11.473 | | 0.009 | 0.010* |
| Isovaleric acid | | | 12.844 | | 0.004 | 0.006** |

P-value was calculated by Kruskal-Wallis test

*FDR q value<0.05

**FDR q value<0.01

| **Mouse cecal sample** | | | **Post-hoc analysis** | | | | | | | | | |
| --- | --- | --- | --- | --- | --- | --- | --- | --- | --- | --- | --- | --- |
|  | **NC/WD** | | | **LL/WD** | | **PP/WD** | | **NC/LL** | | **NC/PP** | | **LL/PP** |
| Acetic acid | | < 0.001** | | | 0.178 | | 0.047* | | 0.013* | 0.054 | 0.457 | |
| Propionic acid | | < 0.001** | | | 0.219 | | 0.049* | | 0.009** | 0.053 | 0.322 | |
| Butyric acid | | < 0.001** | | | 0.251 | | 0.041* | | 0.007** | 0.064 | 0.248 | |
| Iso-butyric acid | | 0.004** | | | 0.376 | | 0.013* | | 0.015* | 0.468 | 0.060 | |
| Valeric acid | | 0.016* | | | 0.220 | | 0.012* | | 0.173 | 0.815 | 0.194 | |
| Iso-valeric acid | | 0.023* | | | 0.286 | | 0.009** | | 0.145 | 0.627 | 0.054 | |

Post-hoc was performed by Dunn’s test with Benjamini–Hochberg correction

*adjusted p value<0.05

**adjusted p value <0.01

| **Human fecal sample** | | |
| --- | --- | --- |
|  | **p-value** | **FDR** |
| Acetic acid | 0.017 | 0.122 |
| Propionic acid | 0.040 | 0.139 |
| Butyric acid | < 0.001 | 0.006** |
| Iso-butyric acid | 0.122 | 0.233 |
| Valeric acid | 0.024 | 0.100 |
| Iso-valeric acid | 0.256 | 0.335 |

P-value and FDR were calculated based on Student’s *t*-test

*FDR q value<0.05

**FDR q value<0.05

**Supplementary Table 8.** Summary of statistics of indole compounds

| **cecum sample** |  |  | | **Kruskal Wallis test** | | |
| --- | --- | --- | --- | --- | --- | --- |
| **Indole metabolites** | | | **chi squared** | | **p-value** | **FDR** |
| Indole-3-lactic acid  Indole-3-propionic acid  Indole-3-acetic acid  Indole-3-acrylic acid  Indole-3-pyruvic acid  Indole-2-carboxylic acid  Methyl indole-3-acetate  5-Hydroxyindole-3-acetic acid  4-Hydroxyindole | | | 5.316  16.637  10.928  10.641  9.287  13.61  16.175  15.713  7.058 | | 0.150  < 0.001  0.012  0.014  0.025  0.003  0.001  0.001  0.070 | 0.188  0.010*  0.027*  0.030*  0.044*  0.012*  0.010*  0.010*  0.099 |
| **Bile acids** | | | **chi.squared** | | **p-value** | **FDR** |
| Cholic acid  Glycocholic acid  Taurocholic acid  Deoxycholic acid  Taurodeoxycholic acid  Glycodeoxycholic acid  Taurochenodeoxycholic acid  Lithocholic acid | | | 13.974  14.042  13.433  7.980  3.923  2.400  13.928  3.367 | | 0.002  0.002  0.003  0.046  0.269  0.493  0.003  0.338 | 0.012*  0.012*  0.013*  0.071  0.309  0.517  0.012*  0.375 |

P-value was calculated by Kruskal-Wallis test

*FDR q value<0.05

**FDR q value<0.01

| **cecum sample** | **Post-hoc analysis** | | | | | |
| --- | --- | --- | --- | --- | --- | --- |
| **Indole metabolites** | **NC/WD** | **LL/WD** | **PP/WD** | **NC/LL** | **NC/PP** | **LL/PP** |
| Indole-3-propionic acid | 0.001** | 0.219 | 0.074 | 0.013* | 0.044* | 0.504 |
| Indole-3-acetic acid | 0.006** | 0.132 | 0.180 | 0.122 | 0.14 | 0.791 |
| Indole-3-acrylic acid | 0.007** | 0.304 | 0.061 | 0.260 | 0.087 | 0.786 |
| Indole-3-pyruvic acid | 0.819 | 0.293 | 0.035* | 0.280 | 0.060 | 0.362 |
| Indole-2-carboxylic acid | 0.007** | 0.496 | 0.594 | 0.013* | 0.014* | 0.995 |
| Methyl indole-3-acetic acid | 0.001** | 0.184 | 0.055 | 0.019* | 0.067 | 0.484 |
| 5-Hydroxyindole-3-acetic acid | 0.001** | 0.219 | 0.139 | 0.019* | 0.026* | 0.729 |
| **Bile acids** | **NC/WD** | **LL/WD** | **PP/WD** | **NC/LL** | **NC/PP** | **LL/PP** |
| Cholic acid | 0.954 | 0.147 | 0.010* | 0.148 | 0.007** | 0.200 |
| Glycocholic acid | 0.002** | 0.040* | 0.245 | 0.208 | 0.034* | 0.261 |
| Taurocholic acid | 0.003** | 0.192 | 0.035* | 0.054 | 0.265 | 0.283 |
| Deoxycholic acid | 0.130 | 0.049* | 0.048* | 0.557 | 0.679 | 0.724 |
| Taurochenodeoxycholic acid | 0.002** | 0.148 | 0.106 | 0.058 | 0.058 | 0.806 |

Post-hoc was performed by Dunn’s test with Benjamini–Hochberg correction

Post-hoc was conducted using only significantly different metabolites from Kruskal wallis test

*adjusted p value<0.05

**adjusted p value <0.01

| **Human fecal sample** | | |
| --- | --- | --- |
| **Indole metabolites** | **p-value** | **FDR** |
| Indole-3-propionic acid | < 0.001 | 0.012* |
| Indole-3-acetic acid | 0.454 | 0.530 |
| Indole-3-acrylic acid | 0.656 | 0.690 |
| Indole-3-lactic acid | 0.180 | 0.291 |
| **Bile acids** | **p-value** |  |
| Cholic acid | 0.019 | 0.099 |
| Glycocholic acid | 0.213 | 0.298 |
| Taurocholic acid | 0.122 | 0.256 |
| Taurodeoxycholic acid | 0.082 | 0.191 |
| Chenodeoxycholic acid | 0.081 | 0.213 |
| Taurochenodeoxycholic acid | 0.158 | 0.276 |

P-value and FDR were calculated based on Student’s *t*-test

*FDR q value<0.05

**FDR q value<0.01
